# Protocol for the functional evaluation of genetic variants using saturation genome editing

**DOI:** 10.1016/j.xpro.2025.103710

**Published:** 2025-04-08

**Authors:** Sofia Obolenski, Rebeca Olvera-León, Dijue Sun, David J. Adams, Andrew J. Waters

**Affiliations:** 1 Experimental Cancer Genetics, Wellcome Sanger Institute, Wellcome Trust Genome Campus, Hinxton, CB10 1SA Cambridge, UK; 2 Department of Dermatology, Leiden University Medical Centre, 2333 ZA Leiden, the Netherlands

**Keywords:** CRISPR, Genetics, Genomics, High-Throughput Screening

## Abstract

Saturation genome editing (SGE) employs CRISPR-Cas9 and homology-directed repair (HDR) to introduce exhaustive nucleotide modifications at specific genomic sites in multiplex, enabling the functional analysis of genetic variants while preserving their native genomic context. Here, we present a protocol for SGE-based variant evaluation in HAP1-A5 cells. We describe the steps for designing variant libraries, single-guide RNAs (sgRNAs), and oligonucleotide primers for PCR. We also detail the sample preparation before the SGE screen, the cellular screening process, and subsequent next-generation sequencing (NGS) library preparation.

For complete details on the use and execution of this protocol, please refer to Radford et al.,^1^ Waters et al.,^2^ and Olvera-León et al.^3^

## Highlights

• SGE protocol in HAP1-A5 cells to assess variant effects on cell fitness over time
• Description of SGE library and sgRNA design, cloning, and HAP1-A5 cell culture
• Steps for SGE screen and SGE-edited gDNA library preparation for NGS
• SGE approach generates functional scores for all SNVs and key variants in CDS, intron, and UTRs

## Abstract (editor highlights)

Publisher’s note: Undertaking any experimental protocol requires adherence to local institutional guidelines for laboratory safety and ethics.

## Graphical abstract

**Figure:**
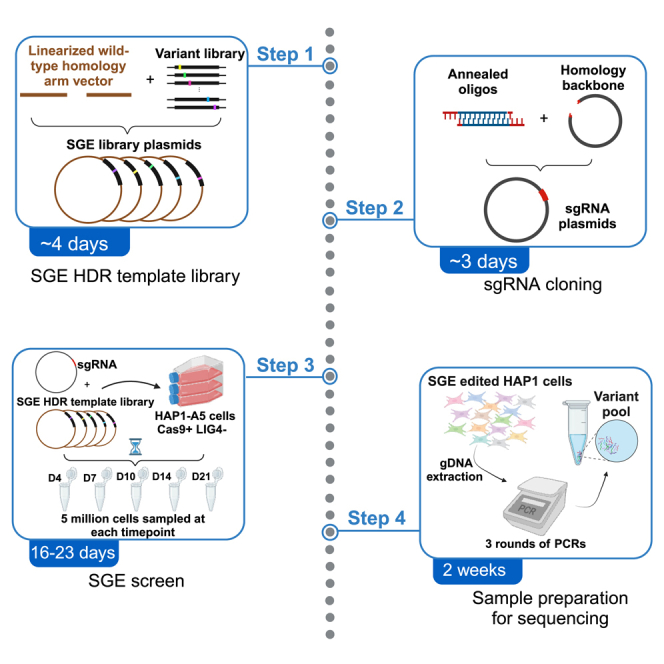

## Before you begin

In the following protocol, we describe the laboratory experiments required to perform saturation genome editing (SGE) to study variant effects at scale on genes that are essential in HAP1-A5 cells (HZGHC-*LIG4*-Cas9). HAP1-A5 is an adherent, near-haploid cell line derived from the KBM-7 chronic myelogenous leukemia cell line.^4^^,^^5^ It carries a DNA Ligase 4 (*LIG4*) gene knockout (KO) generated via a 10 base pair (bp) deletion within the gene, which biases DNA double-strand break repair away from non-homologous end joining (NHEJ) towards the desired homology-directed repair (HDR).^2^ HAP1-A5 cells also have a genomic Cas9 integration, resulting in high Cas9 activity and expression.^2^ This integration ensures high editing efficiency, as Cas9 activity and expression are not limiting.^2^^,^^6^ A key advantage of HAP1-A5 cells is their stable maintenance of haploidy,^2^ which is important for cell fitness readouts in functional genome editing screens. Haploidy at a locus allows recessive phenotypes to manifest with single-allele editing, reducing confounding wild-type allele interference. This is particularly appropriate for variants with loss-of-function (LoF) mechanisms, which includes tumor suppressor genes^7^^,^^8^. However, HAP1 cells can increase in ploidy with prolonged cell culture^1^^,^^9^, leading to greater variability at later time points. HAP1-A5 cells sorted for high haploidy exhibit minimal haploidy loss (<3% between thawing and editing, <5% between editing and the final passage in a three-week SGE screen^9^), which is tolerable and unlikely to introduce appreciable noise into screens. Fluorescence-activated cell sorting (FACS) is a critical step to confirm that the cell stocks are haploid^2^^,^^7^. FACS analysis of thawed stocks is also sensible before conducting a large SGE screen.

**CRITICAL:** Prior to following the protocol described below, it is important to establish the essentiality of the target gene within HAP1-A5 cells. This can be achieved via CRISPR-Cas9-mediated KO of the target gene followed by cell counting or colony assays, or by flow cytometry of cells stained with annexin-V and/or DAPI, to assess cellular apoptosis and/or necrosis. Additionally, a dataset^10^ can be used to predict gene essentiality within HAP1 cells the parental line of HAP1-A5 cells; a Bayes Factor of >6 is considered to have a probability of essentiality of 90%.^8^^,^^10^

**Figure 1:**
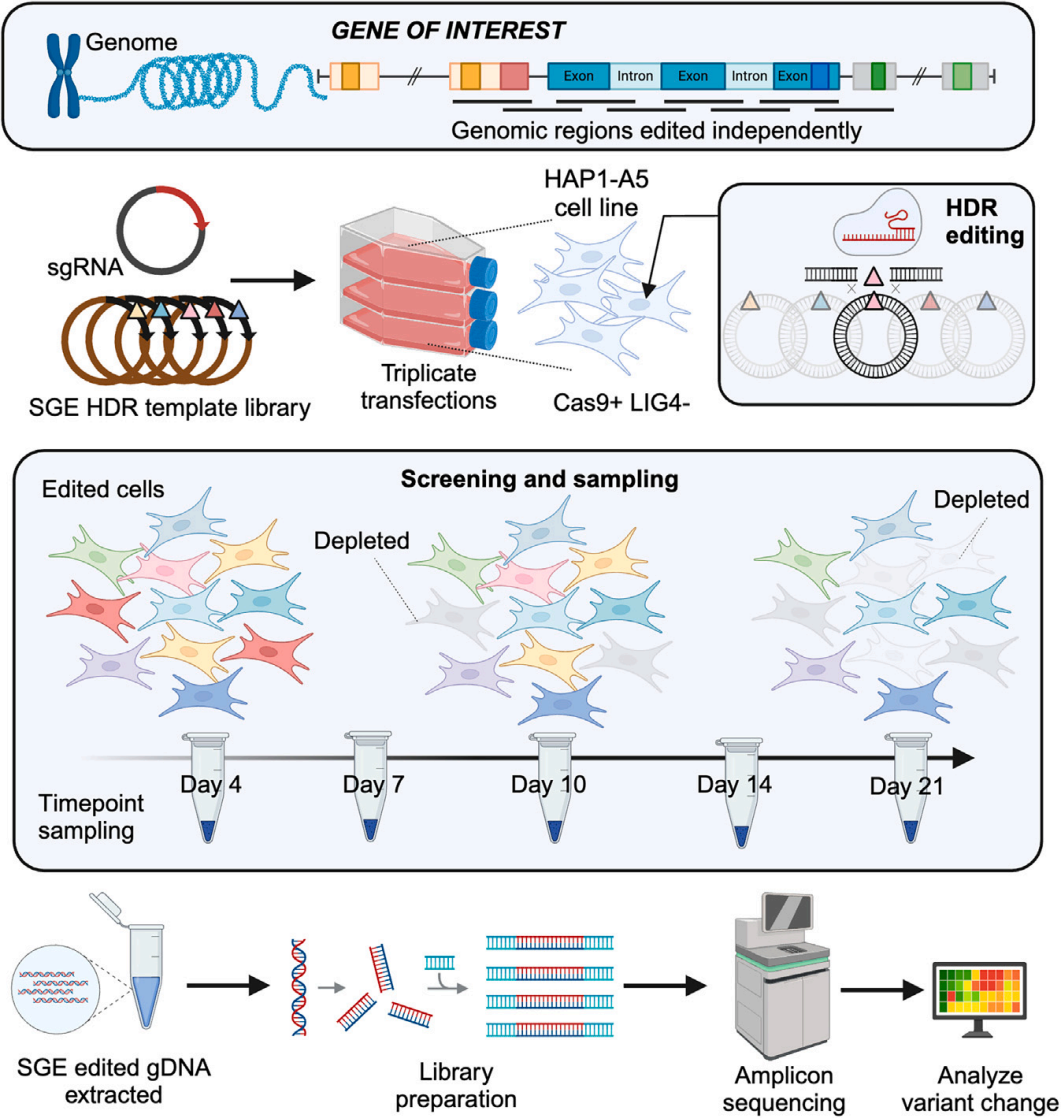
Overview of the complete SGE process workflow In a saturation genome editing (SGE) experiment, each region to be edited in a specific gene is targeted using a single guide RNA (sgRNA) in combination with an SGE homology-directed repair (HDR) template library in HAP1 cells deficient in DNA Ligase 4 (*LIG4*) and expressing endogenous Cas9 (HAP1-A5 cell line). Cas9 induces a double-strand break, which is repaired by the SGE HDR template libraries. Transfections are performed in triplicate, and genomic DNA (gDNA) samples are collected at varying time points depending on the screen length, typically at days 4, 7, 10, 14 and 21. This approach enables functional characterization through variant depletion kinetics, as deleterious alleles in essential loci are expected to decrease over time. The gDNA is then processed for edited gDNA library preparation and sequencing to generate functional scores. This figure is adapted from Olvera-León et al.,^3^ with permission obtained.

An overview of the SGE experimental process is illustrated in Figure 1. As a first step in experimental design, an SGE variant oligonucleotide library is designed together with a corresponding single guide RNA (sgRNA). The SGE variant oligonucleotide library (when cloned into a target-region specific homology region extending plasmid, see below) acts as a repair template, incorporating saturating levels of variation or other desired allele types within target regions of ≤245 bp; each oligonucleotide making up the library contains a discrete variant to be installed into the genome, in addition to synonymous changes at the sgRNA binding site to prevent cleavage of already-installed tracts (we call these fixed changes PAM/protospacer protection edits (PPEs)). SGE variant oligonucleotide library sequences for multiple target regions are then chemically synthesized *en masse* in an oligonucleotide pool*, which is optionally amplified en masse (using **Primer Set 1**) to increase starting material* and then specific target regions within the oligonucleotide pool are amplified using target region-specific **Primer Set 2** (see key resources table), producing 'target specific SGE variant oligonucleotide libraries'. As a second step in this process, the wild-type homology regions of ∼750-1000 bp flanking 5′ and 3′ of the variant containing the target region are generated using **Primer Sets 3 and 4** (key resources table). Subsequently, the wild-type homology region vectors are linearized using **Primer Sets 5** (key resources table) and ligated with the target region specific SGE variant oligonucleotide libraries, resulting in the SGE HDR template libraries.

In addition, appropriate sgRNAs are designed and cloned into a vector containing ampicillin and puromycin resistant cassettes, for selection in *E.coli* and human cells, respectively. The genome for each target region is edited in triplicate by transfecting HAP1-A5 cells with an SGE HDR template library and the corresponding sgRNA, followed by puromycin selection to generate a population of cells, each containing a discrete variant. For genes that are essential in HAP1-A5, variants that compromise the gene function would be expected to be less represented within an edited cell population over time, as these variants result in a loss of fitness. Therefore, cells are cultured and sampled over a time course, with the cell fitness effect of each individual variant analyzed using deep amplicon next-generation sequencing (NGS) libraries. Depending on the kinetics of fitness/degree of essentiality in the target gene and the dynamics of variant effects, edited cells are cultured for a total of 14 or 21 days, on average. Day 4 is the baseline (first) time point collected, with additional time points collected between the baseline and terminal samples, which allows for variant kinetics to be calculated. To maintain good representation of SGE variant installation complexity, 5–6 million cells are collected for each replicate time point. Genomic DNA (gDNA) is then extracted from time point-replicates, and SGE edited gDNA converted to NGS libraries using **Primer Sets 6 – 8** (key resources table). These libraries are then sequenced and analyzed to assess the cell fitness effect of the variants, followed by downstream analyses.

### Source of *LIG4*^-^ HAP1-A5 cells (endogenously expressing CRISPR-Cas9)

The cell line used in the SGE experiment described below can be obtained from Horizon Discovery/Revvity (HAP1-A5 cell line (Lig4- Cas9+ clonal line) (HZGHC-LIG4-Cas9)).

### Institutional permissions

All experiments were conducted with permission from the Wellcome Sanger Institute. Researchers will need to acquire permission from their institution prior to performing these experiments.

### SGE variant oligonucleotide library design

**Timing: 1 week design, vendor-dependent time for ordering SGE variant oligonucleotide library pool**

This section briefly outlines the SGE variant oligonucleotide library design and generation using the software VaLiAnT.^11^

1. Design the SGE variant oligonucleotide library using VaLiAnT^11^:
  a. When editing multiple regions in a gene with SGE, target each region separately.
  b. A target region typically includes a coding sequence exon and adjacent intronic or untranslated regions (UTRs).
    ***Note:*** Due to the ∼300 bp limit of high-quality oligonucleotide synthesis (with a maximum of ∼245 bp reserved for variant edits) larger exons/SGE target regions should be divided into overlapping segments for full SGE coverage.
  c. Define variant-free regions at the target region ends for adding Illumina sequences and to enable targeted PCR amplification from the oligonucleotide pool (one of the reasons why a maximum of ∼245 bp contains variants within a total of ∼300 bp the defined target region in 1.b. above).
    ***Note:*** Variant-free regions should ideally lack polymorphisms or HAP1-A5 specific variants.
  d. Select the desired VaLiAnT^11^ 'mutator' functions for each target region.
    ***Note:*** Libraries may include SNVs, in-frame codon deletions, alanine and stop-codon scans, all possible missense changes (based on a default or user-defined codon usage table), 1 bp deletions, and tandem deletions for splice-site scanning. Custom variants from databases like ClinVar^12^ and gnomAD,^13^ or genome studies, or user-defined indels can also be added as Variant Call Format (VCF) files.
  e. Provide input files, including the genomic coordinates of SGE target regions, gene transfer format (GTF) coding sequence (CDS) annotations, and reference genome sequence into the VaLiAnT^11^ software.
  f. Synthesize oligonucleotide pools via commercial vendors, such as Twist Bioscience.
  g. Resuspend the oligonucleotide pool to 10 ng/μL, following the manufacturer’s instructions.
    i. For Twist bioscience, briefly centrifuge the tube and resuspend in nuclease-free Tris-EDTA (TE) Buffer, pH 8.0.
      ***Note:*** Resuspended oligonucleotide pools can be stored at −20°C for long-term storage.

### sgRNA design

**Timing: 1–2 days, depending on number of target regions**

This section outlines the sgRNA design process^11^ for SGE experiments.

2. Design SGE sgRNAs
  a. For each SGE HDR template library, select a corresponding sgRNA that targets the specific genomic region for editing.
    ***Note:*** Utilize previously reported^11^ selection criteria.
  b. Incorporate synonymous PPEs into each SGE HDR template library target region within the sgRNA binding sites to enhance HDR efficiency; specify PPE coordinates and changes in VCF format for VaLiAnT usage.

## Key resources table

**Table:** 

REAGENT or RESOURCE	SOURCE	IDENTIFIER
**Bacterial and virus strains**		

One Shot TOP10 chemically competent *E. coli*	Thermo Fisher Scientific	Cat#C404010
Endura competent cells	Biosearch Technologies	Item ID: 60242-2

**Chemicals, peptides, and recombinant proteins**		

IDTE pH 8.0 (1X TE solution)	IDT	Cat#11-01-02-05
Exonuclease I (20 U/μL)	Thermo Fisher Scientific	Cat#EN0582
DpnI (20,000 U/mL)	NEB	Cat#R0176S
rCutSmart buffer (10X)	NEB	Cat#B6004S
NEBuilder HiFi DNA assembly master mix (2X)	NEB	Cat#E2621S; Comp#M5520AVIAL
BbsI-HF (20,000 U/mL)	NEB	Cat#R3539S
T4 polynucleotide kinase (10,000 U/mL)	NEB	Cat#M0201L
T4 polynucleotide kinase reaction buffer (10X)	NEB	Cat#B0201S
Quick ligase	NEB	Cat#M2200S
Quick Ligation reaction buffer (2X)	NEB	Cat#B2200S
KAPA HiFi HotStart ReadyMix (2X)	Roche	Kit code: KK2601; Mat.#07958927001
ROX reference dye (%)	Invitrogen	Cat#12223012
EvaGreen dye, 20X in water	Biotium	Cat#31000-T
UltraPure agarose	Thermo Fisher Scientific	Cat#16500500
SYBR safe DNA gel stain	Thermo Fisher Scientific	Cat#S33102
DNA gel loading dye (6X)	Thermo Fisher Scientific	Cat#R0611
HyperLadder 1 kb	Meridian Bioscience	Cat#BIO-33053
S.O.C. (super optimal broth with catabolite repression) medium	Invitrogen	Cat#15544034
Ampicillin (100 mg/mL)	Merck	A5354; Cas#69-52-3
Recovery medium, 8 × 12 mL	Biosearch Technologies	Item ID: 80026-1
repliQa HiFi ToughMix	Quantabio	Mat.#95200-025
Xfect transfection reagent	Takara	Cat#631318
Xfect reaction buffer	Takara	Cat#631318
Trypan blue solution, 0.4%	Gibco	Cat#15250061
Ampure XP	Beckman Coulter	Cat#A63882
Iscove’s modified Dulbecco’s medium (IMDM) (1X)	Thermo Fisher Scientific	Cat#12440-053
Fetal bovine serum (FBS)	Thermo Fisher Scientific	Cat#10437-028
Dulbecco’s phosphate-buffered saline (DPBS)	Sigma-Aldrich	14190144; Cas#38249996
Penicillin-streptomycin (5,000 U/mL)	Gibco	Cat#15070-063
Blasticidin (10 mg/mL)	InvivoGen	Cat#ant-bl-05
Puromycin (10 mg/mL)	InvivoGen	Cat#ant-pr-1
TrypLE express enzyme (1X), phenol red	Gibco	Cat#12605010
0.05% trypsin-EDTA (1x)	Gibco	Cat#25300-054
pmaxGFP vector	NovoPro	Cat#V014332
Sodium acetate buffer solution	Thermo Fisher Scientific	Cat#S7899-100ML
Ampicillin (100 μg/mL) agar plates	N/A	N/A
Lysogenic broth (LB) media	N/A	N/A
50% glycerol	N/A	N/A
Ethanol absolute (100%)	N/A	N/A

**Critical commercial assays**		

QIAquick PCR purification Kit	QIAGEN	Cat#28106
MinElute PCR purification kit	QIAGEN	Cat#28006
QIAprep spin miniprep kit	QIAGEN	Cat#27106
QIAquick gel extraction kit	QIAGEN	Cat#28704
Plasmid plus maxi kit	QIAGEN	Cat#12963
DNeasy blood & tissue kit	QIAGEN	Cat#69506

**Deposited data**		

ClinVar	Landrum et al.^12^	https://www.ncbi.nlm.nih.gov/clinvar/
gnomAD	Karczewski et al.^13^	https://gnomad.broadinstitute.org/

**Experimental models: Cell lines**		

HAP1 cell line (Lig4−)	Horizon Discovery	HZGHC-LIG4
HAP1-A5 cell line (Lig4− Cas9+ clonal line)	Horizon Discovery	HZGHC-LIG4-Cas9

**Oligonucleotides**		

sgRNA oligos	Sigma-Aldrich (100 μM in TE buffer)	N/A
SGE variant oligonucleotide library pool	Twist Bioscience	Lyophilized
Primer Set 1 Generic Illumina adaptor sequences used for SGE variant oligonucleotide library pool amplification (300–350 bp product)	Sigma-Aldrich	Sequence: Illumina_P5: AATGATACGGCGACCACCGA Illumina_P7: CAAGCAGAAGACGGCATACGA
Primer Set 2 Target region-specific primers (∼245–300 bp product)	Sigma-Aldrich	Target-region dependant
Primer Set 3 Primers used to generate the SGE-pMin backbone (1831 bp product)	Sigma-Aldrich	Sequence: SGE_pMin_backbone_F: GCGGCCGCCGTCAGGTGG SGE_pMin__backbone_R: CCTGCAGGACATGTGAG CAAAAGGCCA
Primer Set 4 Primers used for wild-type region amplification (∼1,200–2,000 bp product)	Sigma-Aldrich	Sequence: homology_region _F: TTTTGCTCACATGTCCTGCAGG+ [REGION SPECIFIC SEQUENCE] homology_region _R: GAAAAGTGCCACCTGACG GCGGCCGC+ [REGION SPECIFIC SEQUENCE]
Primer Set 5 Primers used to linearize wild-type homology region plasmid (∼245–300 bp product).	Sigma-Aldrich	Sequence: Linearization_F: Reverse complement sequence of **Primer Set 2** forward Linearization_R: Reverse complement sequence of **Primer Set 2** reverse
Primer Set 6 Primers for sampling/amplification of edited gDNA regions (∼1,700–2,000 bp product)	Sigma-Aldrich	Target-region dependant
Primer Set 7 Primers for sequencing adapter addition/amplification of edited gDNA regions (∼245–300 bp product)	Sigma-Aldrich	Sequence: Sequencing_F: Indexing barcode dependent adaptors +‘**Primer Set 2_F**’ Sequencing_R: Indexing barcode dependent adaptors +‘**Primer Set 2_R**’
Primer Set 8 Indexing primers (∼350 bp product).	Integrated DNA Technologies	N/A
cDNA sequences for colony formation assay	GeneArt	N/A

**Recombinant DNA**		

pMin-U6-ccdb-hPGK-puro (pMin)	Radford et al.^9^	N/A

**Software and algorithms**		

VaLiAnT	Barbon et al.^28^	https://github.com/cancerit/VaLiAnT

**Other**		

Thermocycler	N/A	N/A
Optional: quantitative PCR instrument	N/A	N/A
1.5 mL Eppendorf tubes	N/A	N/A
15 mL conical Falcon tubes	N/A	N/A
50 mL conical Falcon tubes	N/A	N/A
50 mL conical Erlenmeyer flasks	N/A	N/A
500 mL storage bottle (sterile)	N/A	N/A
T150 cell culture flasks	N/A	N/A
T75 cell culture flasks	N/A	N/A
5, 10, 25, and 50 serological pipettes	N/A	N/A
Waterbath (for bacterial work & sterile for cell culture work)	N/A	N/A
Centrifuge	N/A	N/A
Vortex	N/A	N/A
Optional: Countess 3 automated cell counter	Thermo Fisher Scientific	N/A
Optional: Countess cell counting chamber slides and holder, disposable	Thermo Fisher Scientific	Cat#C10312
L-shaped spreaders	Medline Scientific	Cat#MLSP25
Inoculating loops for microbiology	N/A	N/A
5% CO_2_ incubator		
50 mL reagent reservoir	N/A	N/A
96-well cell culture cluster (flat bottom)	N/A	N/A
DynaMag-96 bottom magnet	Thermo Fisher Scientific	Cat#12332D
Optional: Gene Pulser Xcell electroporation systems	Bio-Rad	Cat#1652660
Optional: Gene Pulser/MicroPulser electroporation cuvettes, 0.1 cm gap	Bio-Rad	Cat#1652083
Optional: GelDoc Go imaging system	Bio-Rad	Cat#12009077EDU
Optional: NanoDrop	N/A	N/A
(Shaking) incubator	N/A	N/A

## Materials and equipment

**Table:** Reaction setup for ExoI enzymatic cleanup of the PCR product of Primer Set 1

Reagent	Final concentration	Amount
PCR product of Primer Set 1	N/A	50 μL
Exonuclease I (20 U/μL)	1 U/μL	5 μL
10X Reaction Buffer	1 X	10 μL
ddH_2_O	N/A	35 μL
Total	N/A	100 μL

Keep on ice (0°C–4°C) during use, prepare fresh, and use immediately; do not store.

**Table:** Reaction setup for DpnI digestion of the PCR product of Primer Set 3

Reagent	Final concentration	Amount
PCR product of Primer Set 3 template	N/A	25 μL
DpnI (20,000 U/mL)	0.4 U/μL	1 μL
rCutSmart Buffer (10 X)	1 X	5 μL
ddH_2_O	N/A	19 μl
Total	N/A	50 μL

Keep on ice (0°C–4°C) during use, prepare fresh, and use immediately; do not store.

**Table:** Reaction setup for ligation of wild-type homology region plasmids

Reagent	Final concentration	Amount
PCR product of Primer Set 3	50 ng	X μL
PCR product of Primer Set 4	50 ng	X μL
NEBuilder HiFi DNA Assembly Master Mix (2X)	1 X	10 μL
ddH_2_O	N/A	Complete to 20 μL
Total	N/A	20 μL

Store at −20°C for up to 1 year.

**Table:** Reaction setup for DpnI digestion of the PCR product of Primer Set 5

Reagent	Final concentration	Amount
PCR product of Primer Set 5	N/A	25 μL
DpnI (20,000 U/mL)	0.4 U/μL	1 μL
rCutSmart Buffer (10 X)	1 X	5 μL
ddH_2_O	N/A	19 μL
Total	N/A	50 μL

Keep on ice (0°C–4°C) during use, prepare fresh, and use immediately; do not store.

**Table:** Reaction setup for ligation of linearized wild-type homology regions with target-specific SGE variant oligonucleotide library

Reagent	Final concentration	Amount
Target region-specific SGE variant oligonucleotide library (PCR product of Primer Set 2)	50 ng	X μL
linearized wild-type homology regions (PCR product of Primer Set 5)	50 ng	X μL
NEBuilder HiFi DNA Assembly Master Mix (2X)	1 X	10 μL
ddH_2_O	N/A	Complete to 20 μL
Total	N/A	20 μL

Store at −20°C for up to 1 year.

**Table:** Optional negative control: Reaction setup for ligation of the linearized wild-type homology regions (PCR product of Primer Set 5)

Reagent	Final concentration	Amount
linearized wild-type homology regions (PCR product of Primer Set 5)	50 ng	X μL
NEBuilder HiFi DNA Assembly Master Mix (2X)	1 X	10 μL
ddH_2_O	N/A	Complete to 20 μL
Total	N/A	20 μL

Keep on ice (0°C–4°C) during use, prepare fresh, and use immediately; do not store.

**Table:** Digestion setup for DpnI-digested SGE-pMin backbone

Reagent	Final concentration	Amount
pMin-U6-ccdb-hPGK-puro (pMin))	1 μg	2.5 μL
BbsI-HF (20,000 U/ml)	3.33 U/μL	5 μL
rCutSmart Buffer (10 X)	1 X	3 μL
ddH_2_O	N/A	19.5 μL
Total Volume	N/A	30 μL

Store at −20°C for up to 3 years.

**Table:** Reaction setup for phosphorylation and annealing of complementary sgRNA oligos

Reagent	Final concentration	Amount
Oligo 1 (100 μM)	10 μM	1 μL
Oligo 2 (100 μM)	10 μM	1 μL
T4 Polynucleotide Kinase Reaction Buffer (10 X)	1 X	1 μL
T4 Polynucleotide Kinase (10,000 U/ml)	0.5 U/μL	0.5 μL
ddH_2_O	N/A	6.5 μL
Total Volume	N/A	10 μL

Store at −20°C for up to 1 year.

**Table:** Reaction setup for ligation of sgRNA oligos with pMin_gRNA_backbone

Reagent	Final concentration	Amount
gRNA_pMin_backbone (BbsI-digested)	50 ng	X μL
Phosphorylated and annealed oligo duplex (1:200 dilution)	N/A	1 μL
Quick Ligation Reaction Buffer (2 X)	0.5 X	5 μL
Quick Ligase	N/A	0.8 μL
ddH_2_O	N/A	Complete to 10 μL
Total Volume	N/A	10 μL

Store at −20°C for up to 1 year.

**Table:** Cas9-selection media for HAP1-A5 cell line (‘B+ media’)

Reagent	Final concentration	Amount
Iscove’s Modified Dulbecco’s Medium (IMDM) (1X)	N/A	500 mL
Fetal Bovine Serum (FBS)	10%	50 mL
Penicillin-Streptomycin (5,000 U/mL)	1%	5.5 mL
Blasticidin (10 mg/mL)	10 μg/mL	550 μL
Total	N/A	556.05 mL

Store at 4°C for up to 1 month. Before use, warm the medium to 37°C in a water bath.

**Table:** Growth media for HAP1-A5 cell line (‘noAB media’)

Reagent	Final concentration	Amount
Iscove’s Modified Dulbecco’s Medium (IMDM) (1X)	N/A	500 mL
Fetal Bovine Serum (FBS)	10%	50 mL
Penicillin-Streptomycin (5,000 U/mL)	1%	5.5 mL
Total	N/A	555.5 mL

Store at 4°C for up to 1 month. Before use, warm the medium to 37°C in a water bath.

**Table:** sgRNA selection media for HAP1-A5 cell line (‘B+P+ media’)

Reagent	Final concentration	Amount
Iscove’s Modified Dulbecco’s Medium (IMDM) (1X)	N/A	500 mL
Fetal Bovine Serum (FBS)	10%	50 mL
Penicillin-Streptomycin (5,000 U/mL)	1%	5.5 mL
Blasticidin (10 mg/mL)	10 μg/mL	550 μL
Puromycin (10 mg/mL)	3 μg/mL	165 μL
Total	N/A	556.215 mL

Store at 4°C for up to 1 month. Before use, warm the medium to 37°C in a water bath.

**Table:** Reaction setup for ExoI enzymatic cleanup of the PCR product of Primer Set 6

Reagent	Final concentration	Amount
PCR product of Primer Set 6	N/A	40 μL
Exonuclease I (20 U/μL)	1 U/μL	5 μL
10X Reaction Buffer	1 X	10 μL
ddH_2_O	N/A	45 μL
Total	N/A	100 μL

Keep on ice (0°C–4°C) during use, prepare fresh, and use immediately; do not store.

**Table:** Reaction setup for ExoI enzymatic cleanup of the PCR product of Primer Set 7

Reagent	Final concentration	Amount
PCR product of Primer Set 7	N/A	30 μL
Exonuclease I (20 U/μL)	0.61 U/μL	1 μL
10X Reaction Buffer	1 X	3 μL
Total	N/A	33 μL

Keep on ice (0°C–4°C) during use, prepare fresh, and use immediately; do not store.

## Step-by-step method details

### PCR oligonucleotide pool amplification using Primer Set 1

**Timing: 1 day**

In this step, a PCR is performed with the aim to amplify the oligonucleotide pool in bulk (Figure 1A). For this PCR, Illumina adapter sequences are used as Primer Set 1 and are listed in the key resources table of this protocol. The Illumina adapter sequences are appended to all oligonucleotides to perform a generic amplification of the oligonucleotide pool *en masse*, to increase starting oligonucleotide pool material for downstream cloning processes. Note, these Illumina adapter sequences are not used for sequencing in this step, they are used becuase they perform efficient, unbiased amplifications.

1. Amplify and increase the starting DNA concentration of the oligonucleotide pool via PCR. 3-4 reactions should be independently run and then pooled.
  a. Prepare a PCR reaction master mix as indicated below.
    **Table:** PCR reaction master mix for amplification of the SGE variant oligonucleotide library—Using Primer Set 1

Reagent	Amount
Oligonucleotide pool template	X μL (20 ng)
Illumina_P5 (10 μM)	1.5 μL
Illumina_P7 (10 μM)	1.5 μL
KAPA Hifi HotStart ReadyMix 2x	25 μL
ddH_2_O	Complete to 50 μL
  b. Run a PCR following the conditions below.
    **Table:** PCR cycling conditions for amplification of the oligonucleotide pool—Using Primer Set 1

Steps	Temperature	Time	Number of cycles
Initial Denaturation	95°C	3 min	1
Denaturation	98°C	20 s	12 cycles
Annealing	60°C	15 s	
Extension	72°C	30 s	
Final extension	72°C	30 s	1
Hold	4°C	forever
2. Perform an enzymatic PCR cleanup with Exonuclease (Exo) I (to remove ssDNA & primers).
  a. Prepare a master mix, as described in the materials and equipment section under “Reaction setup for ExoI enzymatic cleanup of the PCR product of Primer Set 1”.
  b. Vortex and mix the reaction.
  c. In a thermocycler, incubate the reaction at 37°C for 20 min followed by 80°C for 20 min.
3. Purify the ExoI-digested PCR product(s) of Primer Set 1 via MinElute columns (QIAGEN), following the manufacturer’s instructions.
  a. Elute the DNA in 10 μL EB Buffer. Pool independent reactions together.
  b. Measure the DNA concentration of the purified PCR product(s) of Primer Set 1.

**Pause point:** The PCR product can be stored at −20°C for long-term storage.

### Primer design for SGE target region amplification—Primer Set 2

**Timing: 30 min, depending on number of target regions**

**Figure 2:**
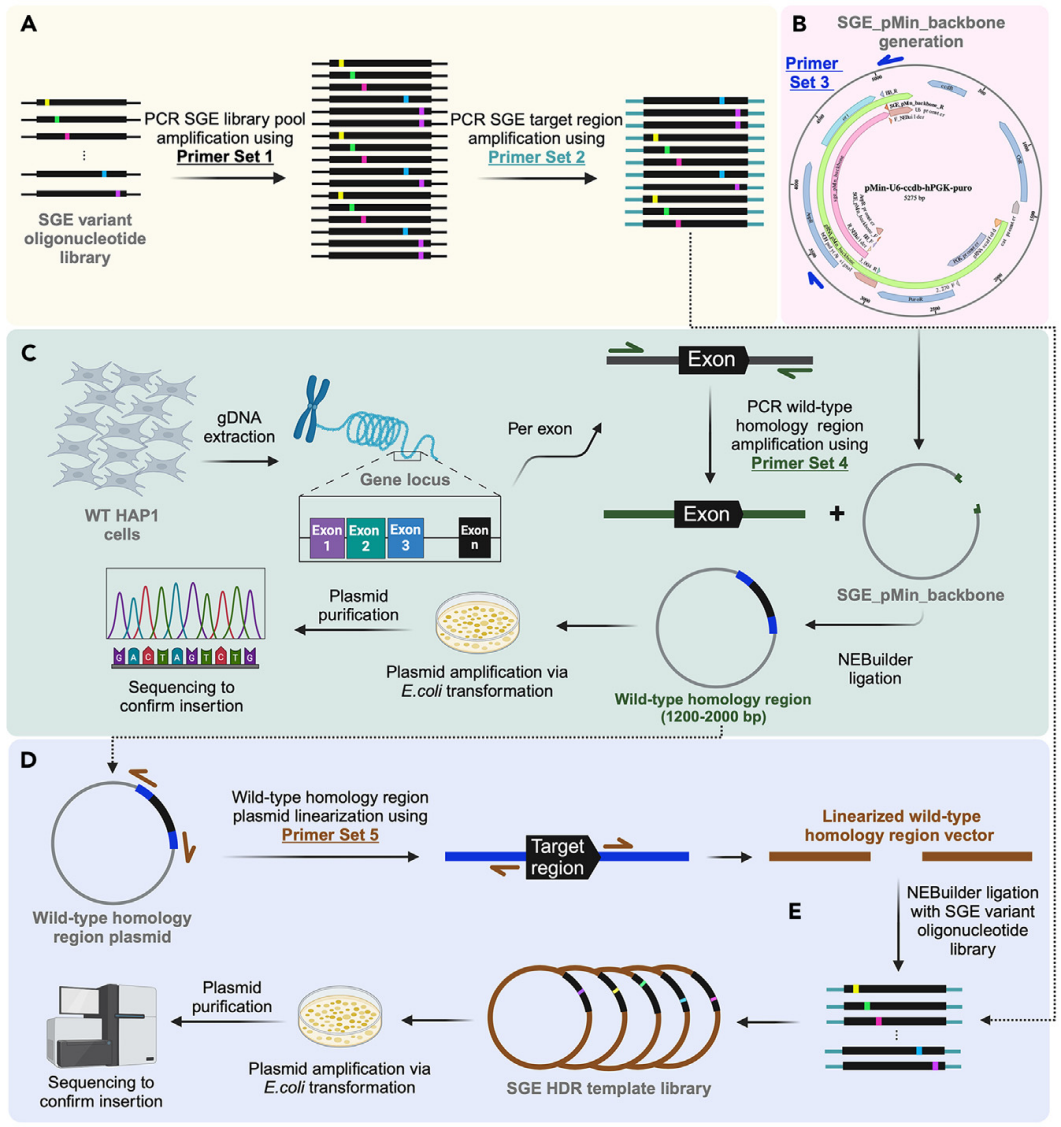
Overview of SGE pre-screen process: Cloning SGE variant oligonucleotide libraries into linearized wild-type homology regions (A) The saturation genome editing (SGE) variant oligonucleotide library pool is PCR amplified using Primer Set 1 to increase the starting material of the SGE variant oligonucleotide library for subsequent cloning. Target region-specific SGE variant oligonucleotide libraries are generated by amplifying the SGE target region using ‘Primer Set 2’. (B) SGE_pMin_backbone is generated using Primer Set 3, which amplifies the promoter and coding sequence for ampicillin resistance as well as the origin of replication (ori) sequence from the pMin-U6-ccdb-hPGK-puro (pMin) plasmid. (C) Wild-type homology arms are amplified from HAP1-A5 genomic DNA (gDNA) using Primer Set 4, with one set designed for each exon. The resulting PCR amplicons are then ligated into the SGE_pMin_backbone, which contains homology regions. The wild-type homology region plasmids are then amplified via *E. coli* transformation and verified by Sanger sequencing. (D) Wild-type homology region plasmids are linearized using Primer Set 5, with specific primers for each target region. (E) The resulting linearized vectors are ligated with the individual target region-specific SGE library oligos to create SGE homology-directed repair (HDR) template libraries. These libraries are amplified via *E. coli* transformation and verified by next-generation sequencing.

This step describes the primer design for the second PCR of the protocol, which aims to amplify the target region-specific sequences from the SGE variant oligonucleotide library pool (product of Primer Set 1) (Figure 2A) using Primer Set 2 as shown in the key resources table of this protocol.

4. Design Primer Set 2 using any preferred primer design software.
  ***Note:*** In this protocol, we used the ‘Primer Design’ function within Geneious Prime, a modified version of Primer 3.
  a. The primers are target region-specific and should define the boundaries of each target region.
  b. The expected product size should range from 245-300 bp.
    ***Note:*** The total length should not exceed 300 bp, when including the Illumina P5/P7 adaptors, due to synthesis limitations (≤300 bp).
  c. When designing primers for multiple target regions, maintain similar product sizes across all targets to ensure consistency and optimal amplification conditions.

### PCR SGE target region amplification using Primer Set 2

**Timing: 1–2 days, depending on number of target regions**

This step outlines the PCR protocol using Primer Set 2, as detailed in the key resources table of this protocol, to amplify specific target regions from the oligonucleotide pool (product of Primer Set 1) (Figure 2A). Prior to the PCR, we recommend performing an optional gradient PCR (e.g., at 58°C, 60°C, 63°C, and 65°C) to determine the optimal annealing temperature for each target region (steps not described) and an optional qPCR (outlined below) to determine the optimal number of amplification cycles, ensuring library complexity is maintained and avoiding over- or under-amplification . If the optional (q)PCR steps are skipped, proceed directly to step 8 of this protocol.

5. **Optional step:** Prepare a qPCR reaction master mix of the PCR product of Primer Set 1 using Primer Set 2 as indicated below.

***Note:*** ROX dye is used as an internal reference; however, alternative dyes as well as varying dye concentrations can be used depending on the qPCR instrument employed. Individual reactions should be set up for each primer pair.

**Table:** qPCR reaction master mix for amplification of the PCR product of Primer Set 1—Using Primer Set 2

Reagent	Amount
PCR product of Primer Set 1 template	X μL (2 ng)
target_region_F (10 μM)	0.45 μL
target_region_R (10 μM)	0.45 μL
KAPA Hifi Hotstart ReadyMix 2x	7.5 μL
EvaGreen (5%)	0.75 μL
ROX (1%)	0.3 μL
ddH_2_O	Complete to 15 μL

6. **Optional step:** Run a qPCR following the conditions below.

**Table:** qPCR cycling conditions for amplification of the PCR product of Primer Set 1—Using Primer Set 2

Steps	Temperature	Time	Number of cycles
Initial Denaturation	95°C	3 min	1
Denaturation	98°C	20 s	35 cycles
Annealing	58°C–65°C	15 s	
Extension	72°C	30 s	
Final extension	72°C	30 s	1
Hold	4°C	forever

7. **Optional step:** Analyze qPCR results.
  a. Identify the most optimal amplification cycle (to maintain library complexity and avoid over/under amplification^6^^,^^14^^,^^15^^,^^16^).
    ***Note:*** An example of a qPCR amplification of 4 target regions is indicated in Figures 3A–3D.

**Figure 3:**
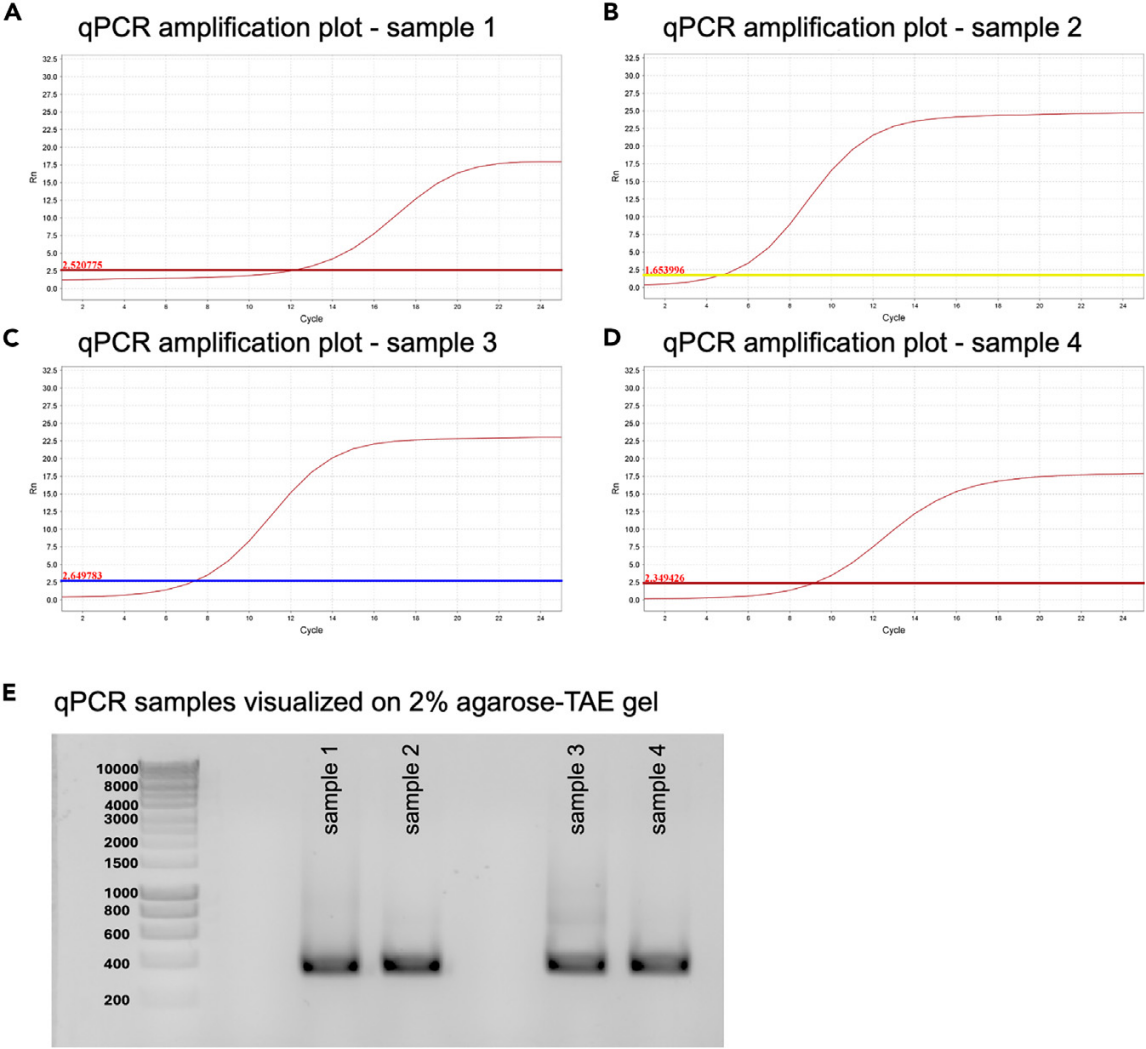
Optimization of qPCR conditions to identify optimal amplification cycles for target-specific primers (A–D) qPCR amplification plots for samples 1–4, illustrating the exponential phase of amplification. The optimal cycle number is determined by identifying the point within the exponential phase prior to the onset of the non-exponential plateau phase. Based on the amplification curves, the optimal cycle numbers for maintaining saturation genome editing (SGE) variant oligonucleotide library complexity and avoiding over- or under-amplification are 19, 11, 13, and 15 cycles for samples 1, 2, 3, and 4, respectively, y-axis is normalized reporter (EvaGreen) fluorescence (Rn) and x-axis is cycle number, the theshold is also displayed by horizontal coloured line. (E) Agarose gel electrophoresis (2% agarose in TAE buffer) of qPCR products after 35 cycles. The expected band size (∼350 base pair (bp)) is observed with appropriate band intensity and no evidence of non-specific amplification.
  b. To assess the purity of the qPCR products a melting curve analysis can be performed.
  c. Run 5 μL of the qPCR product of Primer Set 2 and 2 μL of loading dye on a 2% agarose-TAE gel (30 min at 120 V) to check for correct fragment size (∼245-300 bp), band intensity and potential non-specific bands that may have been co-amplified during the PCR (Figure 3E).
8. Extract and amplify 'target specific SGE variant oligonucleotide libraries' from the oligonucleotide pool via PCR.
  a. Prepare a PCR reaction master mix as indicated below.
    **CRITICAL:** The PCR steps for each exon / target region are performed in separate experiments.
    ***Note:*** Apply the identified amplification cycle number (via qPCR, optional) and annealing temperature (via gradient PCR, optional).
    **Table:** PCR reaction master mix for amplification of the PCR product of Primer Set 1—Using Primer Set 2

Reagent	Amount
PCR product of Primer Set 1 template	X μL (40 ng)
target_region_F (10 μM)	1.5 μL
target_region_R (10 μM)	1.5 μL
KAPA Hifi Hotstart ReadyMix 2x	25 μL
ddH_2_O	Complete to 50 μL
  b. Run a PCR following the conditions below.
    **Table:** PCR cycling conditions for amplification of the PCR product of Primer Set 1—Using Primer Set 2

Steps	Temperature	Time	Number of cycles
Initial Denaturation	95°C	3 min	1
Denaturation	98°C	20 s	10-15 cycles
Annealing	58°C–65°C	15 s	
Extension	72°C	30 s	
Final extension	72°C	30 s	1
Hold	4°C	forever
9. Purify the PCR product(s) of Primer Set 2 via MinElute columns (QIAGEN), following the manufacturer’s instruction.
  a. Measure the DNA concentration of the purified PCR product(s) of Primer Set 2.

### PCR for SGE_pMin_backbone generation using Primer Set 3

**Timing: 1 day**

**Figure 4:**
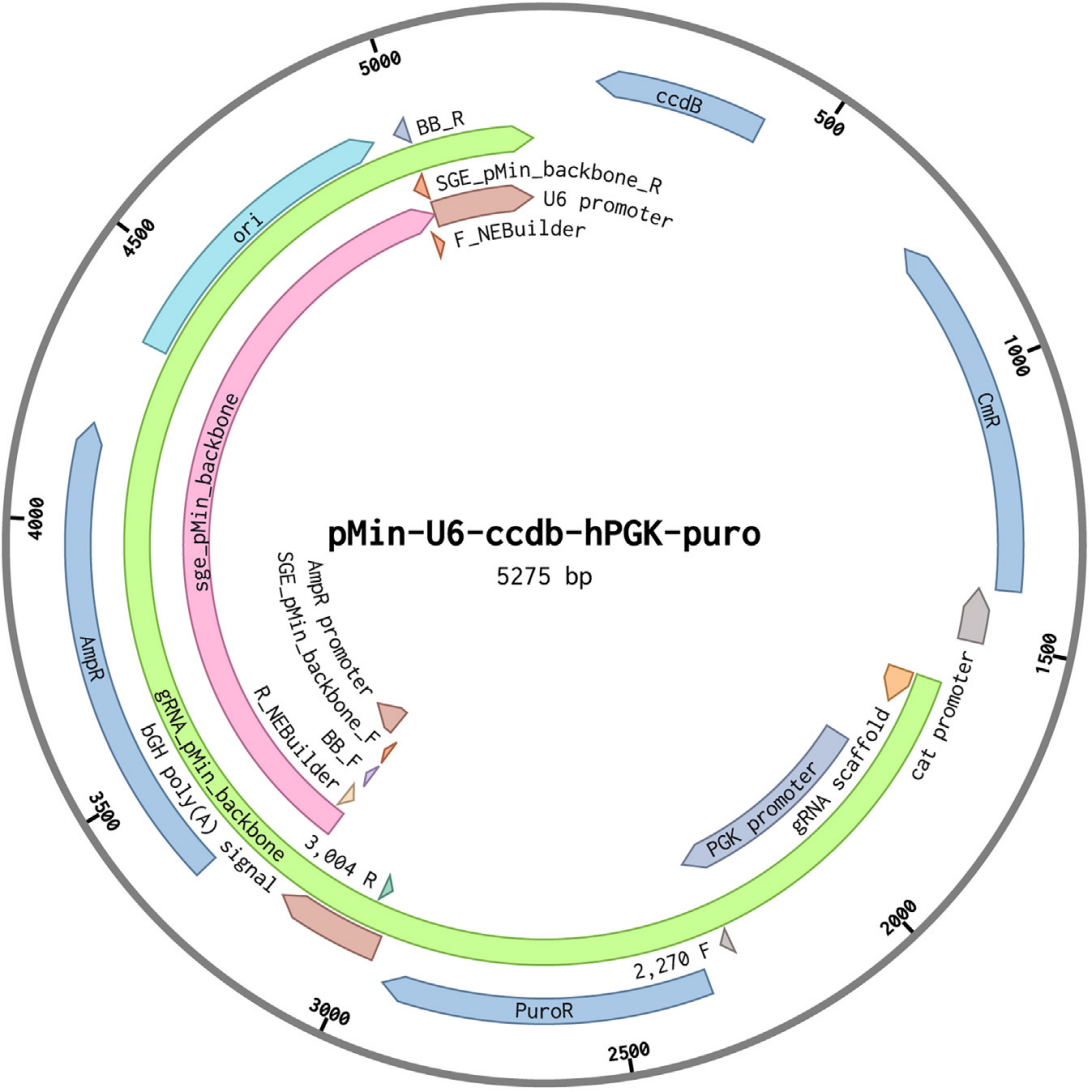
Schematic representation of the pMin-U6-ccdb-hPGK-puro plasmid The pMin plasmid, with a total length of 5.275 kb, serves as the backbone for incorporating the wild-type homology arms (indicated by the pink annotation). Primer Set 3 is employed to amplify the ampicillin resistance (AmpR) gene and the origin of replication (ori) sequence. Additionally, the ‘pMin’ backbone is utilized to construct the ‘gRNA_pMin_backbone’ (green annotation). BbsI-HF restriction enzyme digestion facilitates the inclusion of the AmpR gene, ori sequence, and the puromycin resistance gene, and exclusion of the ccdB casette in downstream clones.

In the step below we generate an SGE_pMin_backbone fragment, which can subsequently be used to create the wild-type homology region plasmids. Use Primer Set 3 here, as listed in the key resources table of this protocol, which anneals to sequences within the pMin plasmid backbone (pMin-U6-ccdb-hPGK-puro (pMin))^1^ and amplifies the backbone regions containing a promoter sequence for ampicillin resistance as well as an origin of replication (*ori*) sequence, 1831 bp in length (Figures 2B and 4).

10. Amplify the SGE_pMin_backbone region from the pMin plasmid backbone.
  a. Prepare a PCR reaction master mix as indicated below.
    **Table:** PCR reaction master mix for generation of SGE_pMin backbone—Using Primer Set 3

Reagent	Amount
pMin plasmid (1:100 dilution of stock)	1 μL
SGE_pMin_backbone_F (10 μM)	0.75 μL
SGE_pMin_backbone_R (10 μM)	0.75 μL
KAPA HiFi Hotstart ReadyMix (2X)	12.5 μL
ddH_2_O	Complete to 25 μL
  b. Run a PCR following the conditions below.
    **Table:** PCR cycling conditions for generation of SGE_pMin backbone—Using Primer Set 3

Steps	Temperature	Time	Number of cycles
Initial Denaturation	95°C	3 min	1
Denaturation	98°C	20 s	25 cycles
Annealing	63°C	15 s	
Extension	72°C	1 min	
Final extension	72°C	1 min	1
Hold	4°C	forever
11. Digest the PCR product of Primer Set 3 with DpnI restriction enzyme (to remove methylated bacterial DNA template, which prevents cloning contamination in subsequent steps).
  a. Prepare a restriction digestion master mix, as described in the materials and equipment section under “Reaction setup for DpnI digestion of the PCR product of Primer Set 3”.
  b. Vortex and mix the reaction.
  c. In a thermocycler, incubate at 37°C for 1 h and subsequently inactivate at 80°C for 20 min.
12. Run the DpnI-digested PCR product of Primer Set 3 on a 1% agarose-TAE gel at 120 V for 30 min.
  ***Note:*** The ‘sge_pMin_backbone’ fragment is 1831 bp long (pink annotation ‘sge_pMin_backbone’ in Figure 4).
  a. Extract the 1831 bp band and purify using QIAquick Gel Extraction Kit (QIAGEN), following the manufacturer’s instructions.
  b. Measure the DNA concentration of the purified PCR product of Primer Set 3.

### Primer design for wild-type homology region amplification—Primer Set 4

**Timing: 30 min, depending on number of target regions**

This step describes the design of Primer Set 4, as listed in the key resources table of this protocol, which is used in a PCR reaction to amplify the wild-type homology sequence on either side of the target region (Figure 2C).

13. Design Primer Set 4 using any preferred primer design software.
  ***Note:*** In this protocol, we used the ‘Primer Design’ function within Geneious Prime, a modified version of Primer 3.
  a. Product size should be between 1200-2000 bp.
    ***Note:*** When designing several target regions, it is recommended to keep the product sizes similar.
  b. The forward primer is 750-1000 bp 5′ of the CDS exon/target region to be saturated with variants and the reverse primer is 750-1000 bp 3’.
  c. The primers define the limits of the target region including homology arms.
  d. The target region to be saturated with variants should roughly central.
  e. For the forward primer, the following adaptor sequence should be added 5’: TTTTGCTCACATGTCCTGCAGG+[SEQUENCE].
    ***Note:*** The adapter sequences are homologous to the amplified fragment of pMin-U6-ccdb-hPGK-puro (pMin)^1^ (DpnI-digested PCR product of Primer Set 3) and are added to the 5′ of the forward primer to allow for NEB Builder cloning.
  f. For the reverse primer, the following adaptor sequence should be added 5’: GAAAAGTGCCACCTGACGGCGGCCGC+[SEQUENCE].
    ***Note:*** The adapter sequences are homologous to the amplified fragment of pMin-U6-ccdb-hPGK-puro (pMin)^1^ (DpnI-digested PCR product of Primer Set 3) and are added to the 5′ of the reverse primer to allow for NEB Builder cloning.

### PCR for wild-type homology region amplification using Primer Set 4

**Timing: 1 day**

In this step a PCR is performed that uses Primer Set 4, as listed in the key resources table of this protocol, to amplify wild-type homology regions surrounding the SGE target regions from HAP1-A5 gDNA. The PCR product of Primer Set 4 will subsequently be ligated into the plasmid backbone (PCR product of Primer Set 3) to generate the wild-type homology region plasmids.

**CRITICAL:** Extract gDNA from HAP1-A5 wild-type cells. Please refer to **steps 46 – 48** of this protocol.

14. Amplify the wild-type-sequence flanking and inclusive of the target region from HAP1-A5 gDNA.
  a. Prepare a PCR reaction master mix as indicated below.
    **CRITICAL:** The PCR steps for each exon / target region are performed in separate experiments.
    **Table:** PCR reaction master mix for wild-type homology region amplification—Using Primer Set 4

Reagent	Amount
Wild-type HAP1-A5 gDNA	X μL (500 ng)
homology_region_F (10 μM)	1.5 μL
homology_region_R (10 μM)	1.5 μL
KAPA HiFi Hotstart ReadyMix (2X)	25 μL
ddH_2_O	Complete to 50 μL
  b. Run PCR following the below conditions.
    **CRITICAL:** Optimal annealing temperature and cycle number need to be standardized for each primer pair per target region.
    **Table:** PCR cycling conditions for wild-type homology region amplification—Using Primer Set 4

Steps	Temperature	Time	Number of cycles
Initial Denaturation	95°C	3 min	1
Denaturation	98°C	20 s	12–25 cycles
Annealing	58°C–65°C	15 s	
Extension	72°C	2 min	
Final extension	72°C	5 min	1
Hold	4°C	forever
15. Run PCR product(s) of Primer Set 4 on a 1% agarose-TAE gel at 100 V for 30 min.
  a. Extract the corresponding band(s) (∼1200-2000 bp) and purify using QIAquick Gel Extraction Kit (QIAGEN), following the manufacturer’s instructions.
  b. Measure the DNA concentration of the purified PCR product(s) of Primer Set 4.

### Generation of wild-type homology region plasmids using PCR products of Primer Set 3 and Primer Set 4

**Timing: 2 days**

In the following steps, we generate the wild-type homology region plasmid for each SGE target region by ligating the SGE_pMin_backbone (PCR product of Primer Set 3) with wild-type homology regions (PCR product(s) of Primer Set 4).

16. Ligate the SGE_pMin_backbone (PCR product of Primer Set 3) with wild-type homology regions (PCR product(s) of Primer Set 4) via NEBuilder HiFi DNA assembly.
  **CRITICAL:** The PCR steps for each exon / target region are performed in separate experiments
  a. Prepare a NEBuilder ligation master mix, as described in the materials and equipment section under “Reaction setup for ligation of wild-type homology region plasmids”.
  b. In a thermocycler, incubate the reaction at 50°C for 1 h.
  c. Dilute the ligated product 1:4 in ddH_2_O.
17. Clone the wild-type homology region plasmids in *E. coli* One Shot TOP10 Chemically Competent cells.
  **CRITICAL:** Set the water bath to 42°C.
  **CRITICAL:** The cloning steps for each exon / target region are performed in separate experiments.
  a. Place ampicillin (100 μg/mL) agar plates into an incubator set to 37°C.
  b. Thaw a vial of 50 μL TOP 10 cells on ice.
  c. Add 2 μL of the wild-type homology region plasmid 1:4 diluted NEBuilder ligation reaction from **step 16.c** to cells.
  d. Incubate cells on ice for 30 min, heat shock at 42°C for 30 s and incubate on ice for 2 min.
  e. Add 950 μL of Super Optimal broth with Catabolite repression (SOC) medium (stored at 20°C–25°C) and place cells at 37°C for 40 min in a shaking incubator at 210 RPM.
  f. Spread 100 μL of cells on pre-warmed ampicillin (100 μg/mL) agar plates and leave at 37°C for 12–16 h.
  g. Next day, inoculate five colonies into separate 15 mL conical Falcon tubes containing 5 mL of Lysogenic Broth (LB) media (supplemented with ampicillin (100 μg/mL)) and incubate in a shaking incubator at 210 RPM at 37°C for 12–16 h.
  h. Prepare glycerol stock from 12 – 16 h grown cultures. Combine 500 μL culture with 500 μL glycerol (50%), mix well and store at −80°C.
  i. Isolate plasmids from the remaining culture using the QIAprep Spin Miniprep Kit (QIAGEN), following the manufacturer’s instructions.
  j. Measure the DNA concentration of the miniprep DNA product.
  k. Confirm correct insertion of the ‘wild-type_homology_region_plasmid’ for each target region via Sanger sequencing using commercial vendors.

### Primer design for the linearization of wild-type homology region plasmid—Primer Set 5

**Timing: 10 min**

In the section below the design for Primer Set 5, as listed in the key resources table of this protocol, is described. These primers aid in the linearization of the ‘wild-type_homology_region_plasmid’ (generated in step **17** of this protocol), so that the individual target specific SGE variant oligocnucleotide libraries (generated in step 6 of this protocol) can subsequently be cloned into the linearized homology backbone.

18. Design Primer Set 5
  ***Note:*** For the design of this set of primers, no design tool is needed.
  a. Reverse complement the sequences of Primer Set 2. These will be the new sequences of Primer Set 5 primers.
    ***Note:*** In this way, the wild-type homology plasmid will be linearized exactly at the border of the target-specific regions (Figure 2D).

### Linearization of the wild-type homology region plasmid using Primer Set 5

**Timing: 1–2 days, depending on number of target regions**

This step describes a PCR for the linearization of homology region plasmids, which will be used as a backbone to be ligated with the individual target specific SGE variant oligonucleotide libraries, using Primer Set 5, as listed in the key resources table of this protocol, to produce SGE HDR template libraries.

19. Linearize the wild-type homology region plasmids.
  a. Prepare a PCR reaction master mix as indicated below.
    ***Note:*** The PCR steps for each exon / target region are performed in separate experiments.
    **Table:** PCR reaction master mix for wild-type homology region plasmid linearization—Using Primer Set 5

Reagent	Amount
wild-type homology region plasmid	X μL (10 pg)
Linearization_F (10 μM)	1.5 μL
Linearization_R (10 μM)	1.5 μL
KAPA HiFi Hotstart ReadyMix (2X)	25 μL
ddH_2_O	Complete to 50 μL
  b. Run a PCR following the conditions below.
    **Table:** PCR cycling conditions for wild-type homology region plasmid linearization—Using Primer Set 5

Steps	Temperature	Time	Number of cycles
Initial Denaturation	95°C	3 min	1
Denaturation	98°C	20 s	35 cycles
Annealing	55°C–65°C	15 s	
Extension	72°C	2 min 30 s	
Final extension	72°C	1 min	1
Hold	4°C	forever
20. Digest the linearized PCR product(s) of Primer Set 5 with DpnI restriction enzyme (to remove methylated wild-type plasmid template).
  a. Prepare a restriction digestion master mix, as described in the materials and equipment section under “Digestion of PCR product of Primer Set 5 with DpnI”.
  b. In a thermocycler, incubate the reaction at 37°C for 1 h and subsequently inactivate at 80°C for 20 min.
21. Run the digested PCR product(s) of Primer Set 5 on a 1% agarose-TAE gel at 120 V for 30 min.
22. Extract the corresponding band and purify using QIAquick Gel Extraction Kit (QIAGEN), following the manufacturer’s instructions.
23. Measure the DNA concentration of the gel-extracted PCR product(s) of Primer Set 5.

### Cloning target-specific SGE variant oligonucleotide libraries into linearized wild-type homology regions

**Timing: 2 days**

This step describes the cloning step of the amplified target specific SGE variant oligonucleotide libraries (PCR product of Primer Set 2) into linearized wild-type homology regions (PCR product of Primer Set 5) to produce the final SGE HDR template libraries that can subsequently be transfected into HAP1-A5 cells (Figure 2E).

***Note:*** The cloning steps for each exon / target region are performed in separate experiments.

24. Prepare a ligation reaction mix, as described in the materials and equipment section under ‘Reaction setup for ligation of linearized wild-type homology regions with target specific SGE variant oligonucleotide libraries’.
  a. In a thermocycler, incubate the NEBuilder ligation reaction at 50°C for 1 h.

**Pause point:** The NEBuilder ligation can be used directly for bacterial cell transformations or can be stored at −20°C.

25. Endura cell transformation to clone SGE HDR template libraries.
  ***Note:*** In this procedure, we use the Endura Electrocompetent Cells with some alterations to the manufacturer’s instructions, as outlined below. For the electroporation of cells, the Gene Pulser Xcell Electroporation System is used. For further details on materials used, please refer to the key resources table.
  ***Note:*** The transformation steps for each exon / target region are performed in separate experiments.
  ***Note:*** Thaw the recovery media 1–2 h prior to the transformation.
  a. Add 2 μL of the NEBuilder ligation reaction (**from step 24.a**) to 25 μL of Endura cells on ice.
    **CRITICAL:** Stir briefly with pipet tip. Do not pipet up and down to mix, which can introduce air bubbles and warm the cells.
  b. Add the cell / ligation mix carefully to the GenePulser Cuvette (0.1 cm gap).
  c. Electroporate cells at 1800 V (capacity: 10 μF, resistance: 600 Ω).
  d. Add 975 μL of recovery media to each sample. Pipette up and down. Transfer the transformation mixture to a 15 mL conical Falcon tube.
  e. Following electroporation of cells, transformed cells are placed in a shaking incubator at 210 RPM at 37°C for 1 h.
  f. Grow 990 μL of transformed cells in 125 mL of LB media (supplemented with ampicillin (100 μg/mL)) for 12–16 h.
  g. Plate 1% of the transformation reaction (10 μL transformed cells with 90 μL SOC medium) onto ampicillin-containing plates and leave at 37°C for 12–16 h. After 12–16 h incubation, evaluate the cultured plates for adequate colony growth/coverage (>500 colonies = >50x cloning coverage for an SGE variant oligonucleotide library containing 1000 variants) to determine if they are suitable for the next experiments.
  h. **Optional step:** Include a negative control to verify the effectiveness of the DpnI digestion in removing any remaining plasmid.
    i. Plate 2 μL of the gel-extracted and DpnI-digested PCR product of Primer Set 5 (**step 23**) onto ampicillin-containing plates and incubate at 37°C for 12–16 h.
    ii. Since the product from **step 23** is linear and should not contain any plasmid, no colony growth is expected if the DpnI digestion was successful.
  i. **Optional step:** Include a negative control to verify the ligation in **step 24a** was performed correctly.
    i. Prepare a ligation reaction mix, as described in the materials and equipment section under ‘Optional negative control: Reaction setup for ligation of the linearized wild-type homology regions (PCR product of Primer Set 5)’.
    ii. In a thermocycler, incubate the NEBuilder ligation reaction at 50°C for 1 h.
    iii. Plate 2 μL of the NEBuilder ligation reaction onto ampicillin-containing plates and incubate at 37°C for 12–16 h.
    iv. No colony growth should be expected, since the ligation is not expected to succeed due to the non-complementary nature of the linearized wild-type homology regions.
  j. Prepare glycerol stock from 12-16 h grown cultures of transformed cells. 500 μL culture combined with 500 μL glycerol (50%), mix well and store at −80°C.
  k. Extract plasmid DNA from cultures using the QIAGEN Plasmid Plus Maxi Kit (QIAGEN), following the manufacturer’s instructions, to isolate the final ‘SGE HDR template library’.
    i. Elute the plasmid DNA in 200 μL EB Buffer.
  l. Confirm the correct insertion of the ‘SGE HDR template library’ for each target region via NGS.

### sgRNA cloning

**Timing: 2–3 days, depending on number of target regions**

This step describes the cloning of target region-specific sgRNAs into the gRNA_pMin_backbone pMin-U6-ccdb-hPGK-puro,^1^ 3653 bp in length, as illustrated by the green annotation ‘gRNA_pMin_backbone’ in Figure 4. The sgRNA design is explained in Section ‘before you begin’ of this protocol. In this procedure, we use the Zhang Lab General Protocol, with some alterations as outlined below.

**CRITICAL:** Use the same pMin-U6-ccdb-hPGK-puro (pMin))^1^ plasmid as used earlier in this protocol.

26. Digest the pMin-U6-ccdb-hPGK-puro (pMin)) backbone with the BbsI-HF restriction enzyme, as described in the materials and equipment section under “Digestion setup for DpnI-digested SGE-pMin backbone”.
  a. In a thermocycler, incubate the reaction at 37°C for 30 min and subsequently inactivate at 65°C for 20 min.
  b. Run the product on a 1% agarose-TAE gel at 120 V for 30 min.
    ***Note:*** The digest will result in two bands: one smaller at 1599 bp and one larger at 3653 bp. The gRNA_pMin_backbone fragment is the 3653 bp band (green annotation ‘gRNA_pMin_backbone’ in Figure 4).
  c. Extract the ∼3.6 kb band and purify using QIAquick Gel Extraction Kit (QIAGEN), following the manufacturer’s instructions.
  d. Assess the concentration and purity of the extracted DNA.
27. Phosphorylate and anneal complementary oligos, as described in the materials and equipment section under “Reaction setup for phosphorylation and annealing of complementary sgRNA oligos”.
  a. Incubate at 37°C for 30 min, then at 95°C for 5 min and finally ramp down to 25 °C at 5 °C/min.
  b. Dilute the reaction 1:200 in ddH_2_O.
28. Ligate the annealed oligos into the BbsI-digested gRNA_pMin_backbone, as described in the materials and equipment section under “Reaction setup for ligation of sgRNA oligos with pMin_gRNA_backbone”.
  a. In a thermocycler, incubate the reaction at 20°C–25°C for 30 min.
  b. Dilute the ligated products 1:4 in ddH_2_O.

**Pause point:** The ligation can be used for subsequent cell transformations or can be stored at −20°C.

29. Clone the sgRNA plasmids in *E. coli* One Shot TOP10 Chemically Competent cells.
  **CRITICAL:** Set a water bath to 42°C.
  a. Place ampicillin (100 μg/mL) agar plates into an incubator set to 37°C.
  b. Thaw 25 μL of TOP10 cells on ice (5 min–10 min).
  c. Add 1 μL of 1:4 diluted ligation to a vial containing 25 μL of thawed TOP10 cells on ice.
  d. Incubate cells/DNA mixture on ice for 30 min, heat shock at 42°C for 30 s and incubate on ice for 2 min.
  e. Add 125 μL of SOC medium (stored at 20°C–25°C) and leave cells at 37°C for 45 min in a shaking incubator at 210 RPM.
  f. Spread 100 μL of cells on pre-warmed ampicillin (100 μg/mL) agar plates and leave at 37°C for 12–16 h.
  g. Next day, inoculate three colonies into separate 15 mL conical Falcon tubes containing 5 mL of LB media (supplemented with ampicillin (100 μg/mL)) and incubate tubes at 37°C in a shaking incubator for 12–16 h.
  h. After 12–16 h prepare glycerol stock from grown cultures.
    i. 500 μL culture combined with 500 μL glycerol (50%), mix well and store at −80°C.
  i. Isolate sgRNA plasmids from the remaining culture using the QIAprep Spin Miniprep Kit (QIAGEN), following the manufacturer’s instructions.
  j. Sanger sequence them to confirm correct insertion.
  k. After sequencing, re-culture correct clones in 250 mL conical Erlenmeyer Flasks using 5 μL of glycerol stock in 125 mL of LB media (supplemented with ampicillin (100 μg/mL)) and place flasks into a shaking incubator at 210 RPM at 37°C for 12–16 h.
    ***Note:*** Culture every exon/SGE target region in an individual conical Erlenmeyer Flask.
  l. Purify sgRNA plasmids using the QIAGEN Plasmid Plus Maxi Kit (QIAGEN), following the manufacturer’s instructions.
  m. Measure the DNA concentration, hereafter referred to as the ‘sgRNA plasmid.

### Thaw and expand HAP1-A5 cells

**Timing: 6–9 days**

Below we outline the procedure for thawing and expanding HAP1-A5 cells before the SGE screen. HAP1-A5 cells need to be thawed and expanded for 2–3 passages, passaging upon reaching 70–80% confluency.

***Note:*** As per Good Laboratory Practice (GLP) guidelines, is recommended to test HAP1-A5 cells for mycoplasma contamination prior to use in SGE experiments.

30. Thaw vial of haploid-sorted HAP1-A5 cells from liquid nitrogen 8–10 days prior to SGE cell culture experiments.
  a. Prepare fresh ‘B+ media’, as described in the materials and equipment section under “Cas9-selection media for HAP1-A5 cell line (‘B+ media’)”.
  b. Thaw cells in a water bath set to 37°C for 2–3 min.
  c. Add 5 mL of ‘B+ media’ into a 15 mL conical Falcon tube.
    i. Add 1 mL of thawed cell suspension to the 15 mL conical Falcon tube.
    ii. Wash the cryovial with 1 mL of ‘B+ media’ and add to the same 15 mL conical Falcon tube.
  d. Spin the cells at 300 x g for 3 min at 20°C–25°C, aspirate liquid.
  e. Resuspend cell pellet in 1 mL of ‘B+ media’ and add to a T150 flask filled with fresh 30 mL of ‘B+ media’.
31. Expand cells for 7–9 days in T75/T150 flasks in 15/35 mL ‘B+ media’.
  a. Aspirate old media.
  b. Wash cells 2 times with 10 mL of Dulbecco’s phosphate buffered saline (DPBS), aspirate and discard DPBS.
  c. Detach cells from culture surface using TrypLE, following the manufacturer’s instructions.
  d. Add 2–4 mL of ‘noAB media’ to the detached cells.
  e. Transfer the detached cells into a 50 mL conical Falcon tube.
  f. Wash the flasks with 5 mL of ‘B+ media’ and add the wash to the same 50 mL conical Falcon tube.
  g. Split the cell suspension across a suitable number of flasks (depending on the confluency of the flask/number of required transfections). Incubate in T75/T150 flasks in 15/35 mL of fresh ‘B+ media’ at 37°C in a 5% CO2 incubator.
  h. Passage the cells every ∼2 days (upon reaching 70–80% confluency).

### SGE screen

**Timing: 16 or 23 days**

This section outlines the tissue culture procedures performed during the SGE screen. For this experiment standard tissue culture facilities (CL1) are required, including a ducted fume hood, microscope, water bath, and cell counting equipment.

**Figure 5:**
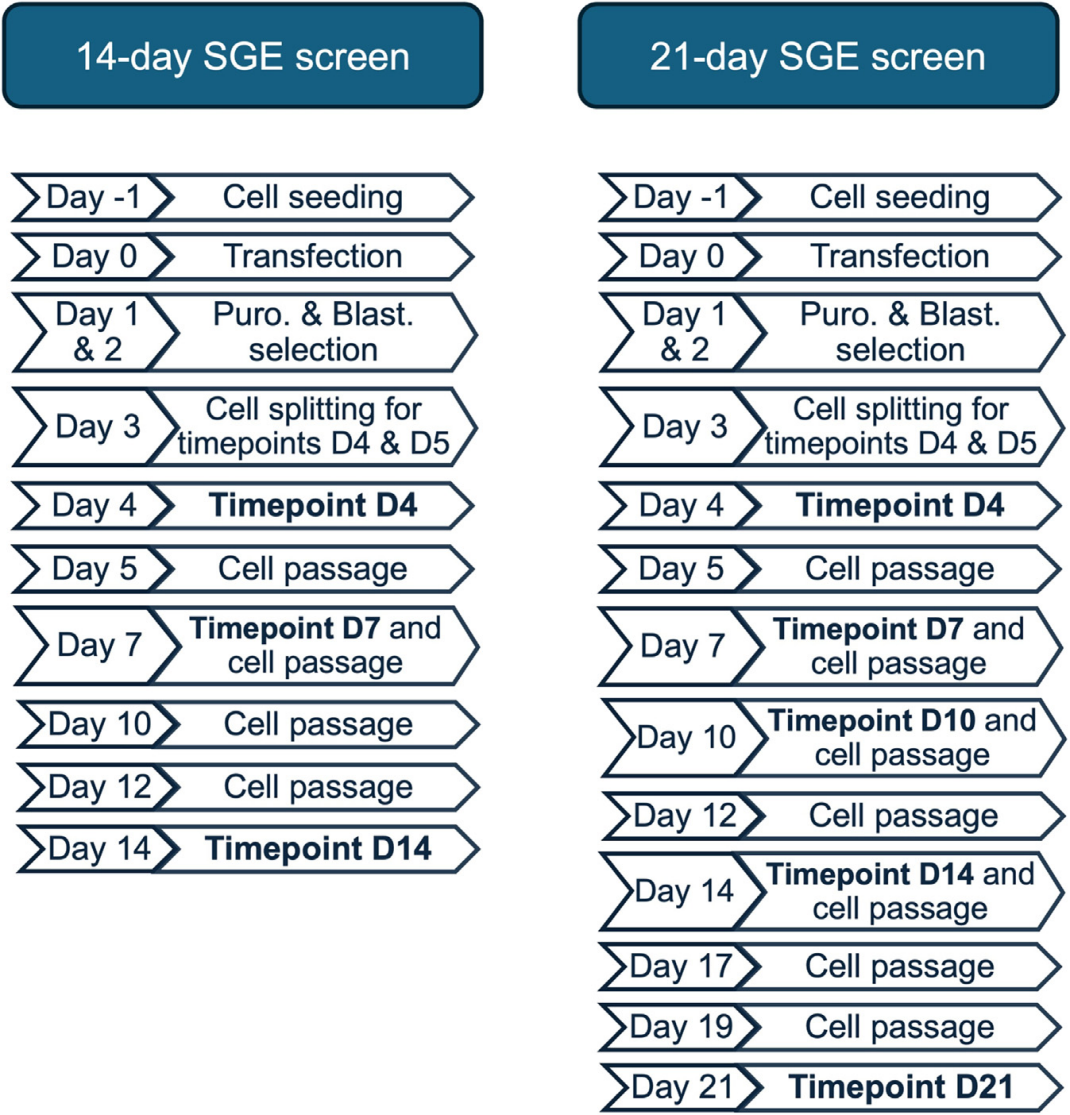
Schematic representation of two SGE tissue culture screening strategies This figure illustrates the two potential saturation genome editing (SGE) screening approaches in tissue culture. For genes with strong essentiality^10^^,^^17^ in the HAP1-A5 cell line, a 14-day SGE screen is appropriate. In contrast, for genes with slower depletion dynamics, a 21-day SGE screen is recommended.

***Note:*** Before initiating the experiments, it is recommended to assess the essentiality of the target gene in HAP1-A5 cells to determine the appropriate screen duration. This assessment can be based on the Bayes Factor^8^^,^^10^ or CRISPR KO screens. For instance, CRISPR KO screens in HAP1-A5 cells can provide insights into the depletion rate of the target gene. Some genes, such as *BAP1*,^2^ may require up to 21 days, whereas others (e.g. *RAD51C*^3^) may be sufficiently analyzed within 14 days (Figure 5). The chosen duration will also dictate the timing of gDNA time point sampling.

32. Day −1: Cell seeding.
  a. Prepare fresh ‘noAB media’, as described in the materials and equipment section under “Growth media for HAP1-A5 cell line (‘noAB media’)”.
  b. Aspirate old media from all flasks you have in culture.
  c. Wash each flask 2 times with 5 mL of DPBS, aspirate and discard DPBS.
  d. Detach cells from culture surfaces using TrypLE, following the manufacturer’s instructions.
  e. Add 2–4 mL of ‘noAB media’ to the detached cells.
    ***Note:*** Add at least the same volume of media as the volume of trypsin used.
  f. Transfer the detached cells into (several) 50 mL conical Falcon tube(s).
    ***Note:*** Depending on the number of flasks, use the appropriate number of conical Falcon tubes. Since the cells are wild-type at this stage, cells from multiple T75 flasks can be pooled into a single 50 mL conical Falcon tube.
  g. Wash each flask with 5 mL of ‘noAB media’ and add the wash to the same 50 mL conical Falcon tube(s).
  h. Count the cells.
    ***Note:*** We use Countess 3 Automated Cell Counter.
    i. Add 10 μL of a 1:1 mixture of trypan blue and the cell suspension.
  i. Make a suspension that contains 8 million cells in 15 mL of ‘noAB media’.
  j. Example calculation (for 3 exons in replicate and 3 control flasks: sgRNA-only transfection, sgRNA + SGE HDR template library transfection, HAP1-A5 wild-type untransfected):
    Calculate the total number of cells needed for all flasks:
    $$11\left(total\;of\;flasks\right)\;x\;\left(8x10^{6}\;/\;concentration\;of\;cells\;per\;ml\;measured\right)=X$$
    Calculate the total volume of media required:
    11(flasks)x15ml(finalvolumeperflask)=165ml
    Subtract the calculated volume of the cell suspension (X) from the total volume:
    165ml−X=mediavolumetoaddtoXtomakestockofcellsuspension
    The remainder is ‘noAB media’.
  k. Seed 15 mL of cell suspension into 12 T75 flasks.
  l. Maintain the cells at 37°C in a 5% CO_2_ incubator.
  m. Thaw Xfect Buffer at +4°C for 16–20 h.
33. Day 0: Transfection.
  a. Leave Xfect Buffer at 20°C–25°C for 1–2 h before starting tissue culture work.
  b. Aspirate old ‘noAB media’ and add 10 mL of fresh ‘noAB’ media. Allow the cells to incubate with fresh media for at least 1 h before transfection.
  c. Prepare the transfection mixture toward the end of the 1 h incubation.
    i. Combine 7.5 μg sgRNA and 15 μG SGE HDR template library per exon/target region in a 1.5 mL Eppendorf tube.
      ***Note:*** Perform the transformation mixtures in biological triplicates per exon / SGE target region sample.
    ii. Prepare the sgRNA-only transfection control sample by adding 7.5 μG sgRNA of a selected sgRNA together with 15 μg of a GFP plasmid to a 1.5 mL Eppendorf tube.
    iii. Add Xfect Buffer to adjust the final volume to 750 μL.
    iv. Vortex the samples briefly.
    v. Thaw the Xfect polymer briefly before adding 13.5 μL to each reaction (calculated as 0.6 μL of Xfect polymer per 1 μg of DNA, with 22.5 μg of DNA requiring 13.5 μL Xfect polymer).
    vi. Vortex each sample and incubate at 20°C–25°C for 10 min.
      **CRITICAL:** Ensure the incubation time does not exceed 30 min.
    vii. Very briefly spin samples at <300 x g for 2-3 s at 20°C–25°C.
  d. After the 1 h incubation add the prepared transfection mixture to the cells.
    i. Position the T75 flask vertically and add the transfection mixture dropwise to the media. Then tilt the flask horizontally and gently swirl the media to ensure even distribution over the cells.
      **CRITICAL:** Maintain the flask horizontally while processing other flasks.
      ***Note:*** Perform the transformation in biological triplicates per exon/SGE target region sample.
      ***Note:*** For the sgRNA-only transfection control, apply the corresponding transfection mixture. Do not add any transfection mixture to the HAP1-A5 wild-type untransfected T75 control flask.
  e. Incubate the flasks at 37°C for 4 h.
  f. Aspirate media and replace with 15 mL of fresh ‘noAB media’.
  g. Leave cells to recover at 37°C in a 5% CO_2_ incubator for 16–20 h.
34. Day 1: Selection.
  a. Aspirate old media and replace with 15 mL of fresh ‘B+P+ media’, as described in the materials and equipment section under “sgRNA selection media for HAP1-A5 cell line (‘B+P+ media’)”.
  b. For the sgRNA-only transfection control: add 15 mL of ‘B+P+ media’.
  c. For the HAP1-A5 wild-type untransfected control: add 15 mL of ‘B+ P+media’, if using as a control for puromycin selection (i.e. to confirm complete cell death is achieved in this control), which is recommeneded to ensure efficient sample selection. If using as a control for wild-type cell growth use 'B+ media'.
  d. Leave cells at 37°C in a 5% CO_2_ incubator for 16–20 h.
35. Day 2: Selection.
  a. Aspirate old media from all T75 flasks and replace with 15 mL of fresh ‘B+P+ media’.
  b. Examine cells under a microscope to assess cell viability.
    ***Note:*** See Figure 6 for representative images of cell populations under ‘B+P+ media’ selection, with and without transfection. The sgRNA-induced effects on cell fitness/death are evident in sgRNA-only transfection control samples, often showing reduced cell density (Figure 6A). In contrast, sgRNA + SGE HDR template library transfection generally enhances survival, with most variants rescuing the fitness phenotype, and resulting in confluency similar to HAP1-A5 wild-type untransfected cells (Figures 6B and 6C). While controls are optional, differences in cell fitness can confirm transfection efficiency, especially in multiple larger experiments (as often some guides will show this effect); in addition, replicates should all show similar effects, which can give a rough indication that experimental accuracy is acceptable.

**Figure 6:**
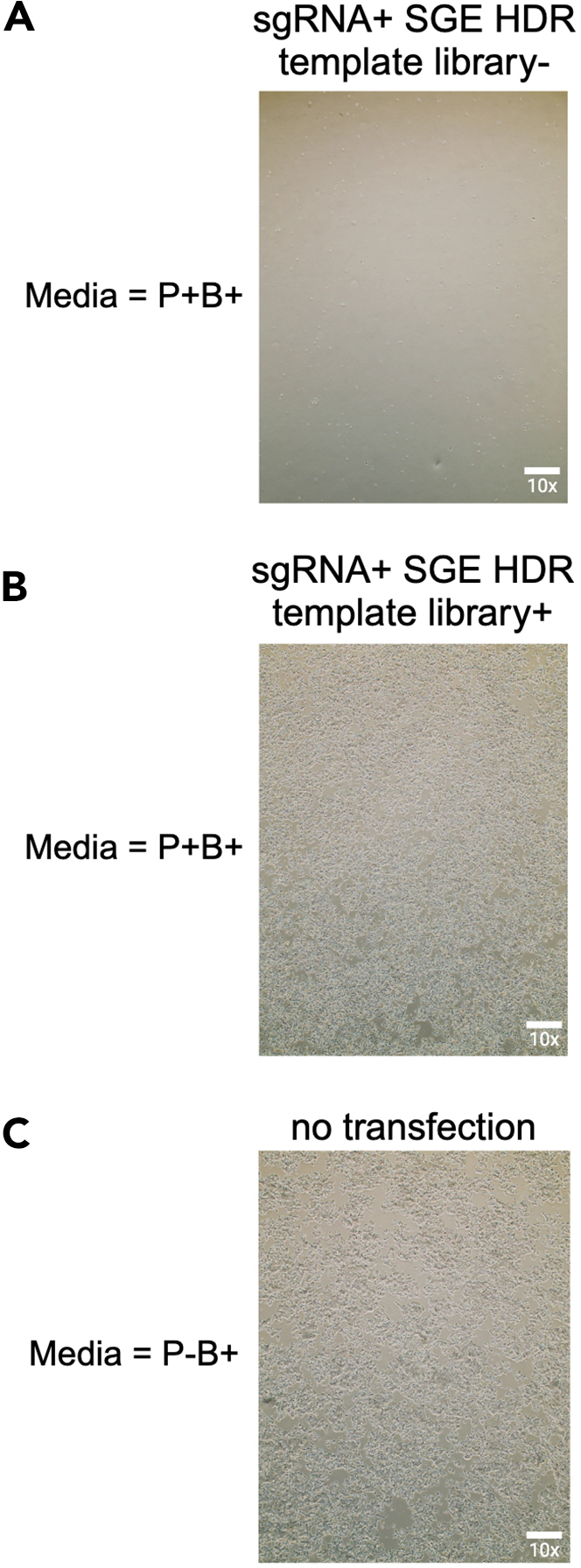
Imaging of HAP1-A5 cells under different tissue culture conditions for the SGE screen (A) HAP1-A5 cells transfected with single guide RNA (sgRNA) alone and selected using puromycin (P+) and blasticidin (B+) media. The efficacy of the sgRNA in inducing a cell fitness or cell death phenotype may result in fewer cells in control samples, where only sgRNAs are transfected. (B) HAP1-A5 cells transfected with both the saturation genome editing (SGE) homology-directed repair (HDR) template library and the corresponding sgRNA, and selected with puromycin (P+) and blasticidin (B+) media, exhibit 70–80% confluency. This is because the SGE HDR template library serves as an HDR template, and most variants in the library rescue the cell fitness phenotype. (C) Untransfected wild-type HAP1-A5 cells also display 70–80% confluency. All images were captured using the Evos XL microscope at 10x magnification (scale bar indicated).
  c. Leave cells at 37°C in a 5% CO_2_ incubator for 16–20 h.
36. Day 3: Split cells for subsequent time points.
  a. Aspirate old media from all T75 flasks.
  b. Wash each flask 2 times with 5 mL of DPBS, aspirate and discard DPBS.
  c. Detach cells from culture surface using TrypLE, following the manufacturer’s instructions.
  d. Add 2–4 mL of ‘B+ media’ to the detached cells.
    ***Note:*** Add at least the same volume of media as the volume of trypsin used.
  e. Transfer the detached cells into a 15 mL conical Falcon tube.
    ***Note:*** Use a separate 15 mL conical Falcon tube for each flask.
  f. Wash the flasks with 5 mL of ‘B+ media’ and add the wash to the same 15 mL conical Falcon tube.
    i. Resuspend the cell mixture gently by pipetting up and down.
  g. Divide the cell suspension of each 15 mL Falcon tube into two equal portions, transferring 50% of cells to one T75 flask and 50% to another.
    ***Note:*** Label one flask with the corresponding sample name followed by ‘Day 4’, and label the other flask with the sample name followed by ‘Day 5’ to indicate the respective time points.
  h. Incubate all T75 flasks in 15 mL of fresh ‘B+ media’, as described in the materials and equipment section under “Cas9-selection media for HAP1-A5 cell line (‘B+ media’)”, and leave at 37°C in a 5% CO_2_ incubator for 16–20 h.
37. Day 4: Time point ‘Day 4’ –gDNA extraction
  a. Aspirate old media from T75 flasks labeled ‘Day 4’.
  b. Wash the T75 flasks with 5 mL of DPBS, aspirate and discard.
  c. Detach cells from culture surface using TrypLE, following the manufacturer’s instructions.
  d. Add 2–4 mL of ‘B+ media’ to the detached cells.
    ***Note:*** Add at least the same volume of media as the volume of trypsin used.
  e. Transfer the detached cells into a 15 mL conical Falcon tube.
    ***Note:*** Use a separate 15 mL Falcon tube for each flask.
  f. Wash each flask with 5 mL of ‘B+ media’ and add the wash to the same 15 mL conical Falcon tube.
  g. Count the cells.
    i. Add 10 μL of a 1:1 mixture of trypan blue and the cell suspension.
  h. Centrifuge the 15 mL Falcon tubes at 300 x g for 3 min.
  i. Carefully aspirate the supernatant and resuspend the cell pellets in DPBS to achieve a concentration of 5–6 million cells per mL.
  j. Aliquot 5–6 million cells per mL into a 1.5 mL Eppendorf tube.
    ***Note:*** It is recommended to aliquot several, at least 3, technical replicates per sample.
  k. Spin the 1.5 mL Eppendorf tubes at 300 x g for 3 min at 20°C–25°C, carefully remove the DPBS, and store pellets at −80°C.
  l. Discard any excess cells.
38. Day 5: Passage cells.
  a. Aspirate old media from T75 flasks labeled ‘Day 5’.
  b. Wash the T75 flasks with 5 mL of DPBS, aspirate and discard.
  c. Detach cells from culture surface using TrypLE, following the manufacturer’s instructions.
  d. Add 2–4 mL of ‘B+ media’ to the detached cells.
    ***Note:*** Add at least the same volume of media as the volume of trypsin used.
  e. Transfer the detached cells into a 15 mL conical Falcon tube.
    ***Note:*** Use a separate 15 mL Falcon tube for each flask.
  f. Wash each flask with 5 mL of ‘B+ media’ and add the wash to the same 15 mL conical Falcon tube.
  g. Count the cells.
    i. Add 10 μL of a 1:1 mixture of trypan blue and the cell suspension.
  h. Centrifuge the 15 mL Falcon tubes at 300 x g for 3 min.
  i. Carefully aspirate the supernatant and resuspend the cell pellets in fresh ‘noAB media’ to achieve a concentration of 5–6 million cells per mL.
  j. Seed 5–6 million cells into one T75 flask in 15 mL of fresh ‘noAB media’.
    ***Note:*** Label each flask with the corresponding sample name followed by ‘Day 7’.
  k. Leave cells at 37°C in a 5% CO2 incubator until ‘Day 7’.
39. Day 7: Time point ‘Day 7’ – extraction of gDNA and cell passage.
  a. Aspirate old media from T75 flasks labeled ‘Day 7’.
  b. Wash the T75 flasks with 5 mL of DPBS, aspirate and discard.
  c. Detach cells from culture surface using TrypLE, following the manufacturer’s instructions.
  d. Add 2–4 mL of ‘B+ media’ to the detached cells.
    ***Note:*** Add at least the same volume of media as the volume of trypsin used.
  e. Transfer the detached cells into a 15 mL conical Falcon tube.
    ***Note:*** Use a separate 15 mL Falcon tube for each flask.
  f. Wash each flask with 5 mL of ‘B+ media’ and add the wash to the same 15 mL conical Falcon tube.
  g. Count the cells.
    i. Add 10 μL of a 1:1 mixture of trypan blue and the cell suspension.
  h. Centrifuge the 15 mL Falcon tubes at 300 x g for 3 min.
  i. Carefully aspirate the supernatant and resuspend the cell pellets in fresh ‘noAB media’ to achieve a concentration of 5–6 million cells per mL.
  j. Seed 5–6 million cells into one T150 flask in 35 mL of fresh ‘noAB media’.
    ***Note:*** Label each flask with the corresponding sample name followed by ‘Day 10’.
  k. Leave cells at 37°C in a 5% CO2 incubator until ‘Day 10’.
  l. Spin the remaining cell suspension, 300 x g for 3 min at 20°C–25°C.
  m. Add 5 mL of DPBS, spin at 300 x g for 3 min at 20°C–25°C.
  n. Carefully aspirate the supernatant and resuspend the cell pellets in DPBS to achieve a concentration of 5–6 million cells per mL.
  o. Aliquot 5–6 million cells per mL into a 1.5 mL Eppendorf tube.
    ***Note:*** It is recommended to aliquot several, at least 3, technical replicates per sample.
    Spin the 1.5 mL Eppendorf tubes at 300 x g for 3 min at 20°C–25°C, carefully remove the DPBS, and store pellets at −80°C.
40. Day 10: Time point ‘Day 10’ – extraction gDNA and cell passage.
  a. Repeat the same steps as for ‘Day 7’ with one modification: seed 5–6 million cells into a T75 flask rather then a T150 flask to provide optimal surface area for 2 days growth rather than 3 days, as the cells will be passaged on ‘Day 12’.
41. Day 12: Passage cells.
  a. Repeat the same steps as for ‘Day 5’.
42. Day 14: Time point ‘Day 14’ – extraction gDNA and cell passage.
  a. Repeat the same steps as for ‘Day 7’.
43. Day 17: Passage cells.
  a. Repeat the same steps as for ‘Day 5’.
44. Day 19: Passage cells.
  a. Repeat the same steps as for ‘Day 5’.
45. Day 21: Time point ‘Day 21’ – extraction gDNA.
  a. Repeat the same steps as for ‘Day 4’.

### gDNA extraction from cell pellets

**Timing: 1–2 days, depending on number of samples**

After completing the SGE tissue culture experiments, gDNA is extracted and prepared for NGS submission. The following section outlines the steps for extracting gDNA from HAP1-A5 cell pellets obtained during the previous SGE screen experiment.

**CRITICAL:** Set a thermocycler to 56°C.

46. Thaw cell pellets for each time point-replicate.
47. Resuspend in 200 μL DPBS.
48. Extract gDNA from each pellet using the DNeasy Blood & Tissue Kit (QIAGEN), following the manufacturer’s instructions, with a few adaptations to the protocol as outlined below.
  ***Note:*** All collection tubes used for the washing steps are provided, except for the final 1.5 mL Eppendorf tubes where the DNA product is eluted.
  a. Add 4 μL of RNAse A (100 mg/mL). Incubate at 37°C for 15 min.
  b. Add 20 μL of Proteinase K.
  c. Add 200 μL of Buffer AL. Mix thoroughly (vortex and a quick spin at 300 x g for 30 s at 20°C–25°C).
  d. Incubate at 56°C for 15 min.
  e. Add 200 μL of Ethanol (96–100%). Mix thoroughly. (vortex and a quick spin at 300 x g for 30 s at 20°C–25°C)
  f. Mix the mixture by pipetting up and down for 5 times. Pipette 700 μL (all) of the mixture into the DNAEasy Mini spin column placed in a 2 mL collection tube (provided within the kit). Centrifuge at 9500 RPM for 1 min.
  g. Place the spin column in a new collection tube. Add 500 μL of Buffer AW1. Centrifuge at 9500 RMP for 1 min. Discard the flow-through and the collection tube.
  h. Place the spin column in a new collection tube. Add 500 μL of Buffer AW2. Centrifuge at 13 200 RPM for 1 min. Discard the flow-through. Centrifuge for another 2 min at 13 200 RPM. Discard the flow-through and the collection tube.
  i. Place the spin column into a new 1.5 mL Eppendorf tube (not provided within the kit).
  j. Elute gDNA in 100 μL of Buffer AE.
    ***Note:*** For samples that have less than 5–6 million cells, elute in 50 μL Buffer AE.
  k. Incubate at 20°C–25°C for 5 min.
  l. Centrifuge at 13 200 RPM for 1 min.
    ***Note:*** The solution should look viscous.
  m. Measure the DNA concentration.
    ***Note:*** From a 5–6 million cells pellet, gDNA concentrations of ≥ 10 μg in 100 μL are to be expected.

### Primer design for PCR for sampling/amplification of edited gDNA regions—Primer Set 6

**Timing: 30 min, depending on number of samples**

The following step outlines the primer design for Primer Set 6, as shown in the key resources table of this protocol. This primer set is used in a PCR to sample/amplify SGE targeted regions from the HAP1-A5 extracted edited gDNA.

49. Design Primer Set 6 using any preferred primer design software.
  ***Note:*** In this protocol, we used the ‘Primer Design’ function within Geneious Prime, a modified version of Primer 3.
  a. Product size should be between 1700-2000 bp.
    ***Note:*** When designing several target regions, it is recommended to keep the product sizes similar.
  b. The Forward primer should be positioned outside the sequence range of Primer Set 4, as shown in the key resources table of this protocol. This ensures that amplification does not occur from any residual SGE HDR template library plasmid present in the gDNA extractions.

### PCR for sampling/amplification of edited gDNA regions using Primer Set 6

**Timing: 2 days**

**Figure 7:**
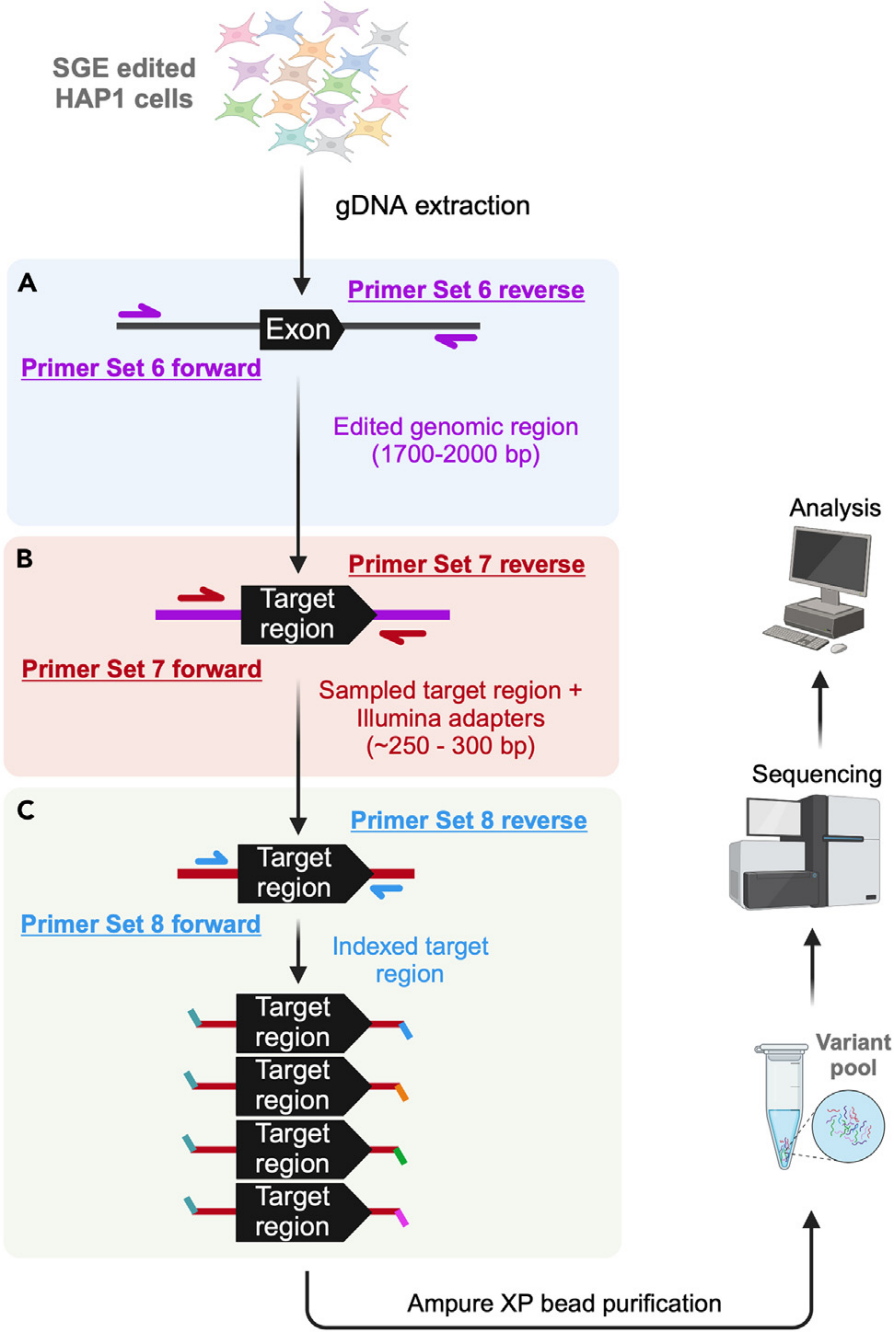
Overview of SGE-edited genomic DNA library samples preparation for sequencing Once the saturation genome editing (SGE) tissue culture experiments are completed, cell pellets are collected, and genomic DNA is extracted to prepare the SGE edited genomic DNA (gDNA) library for next-generation sequencing (NGS). Library preparation includes three rounds of PCR. (A) Primary PCR: Amplification of the genomic region edited by SGE is performed using Primer Set 6, with a specific set of primers designed for each exon. (B) Secondary PCR: To amplify the specific target region from the sample and add Illumina adapters, Primer Set 7 is used. (C) Indexing PCR: To add unique barcodes to all samples for NGS (including replicates and time points), Primer Set 8 is used for each individual sample. After indexing, all samples are bead-purified, pooled and sequenced for subsequent analysis.

In this step, a PCR is performed using Primer Set 6, as shown in the key resources table of this protocol, to amplify the edited SGE genomic region from the collected gDNA pool. Please refer to Figure 7A for an illustration of this process.

***Note:*** Before performing the PCR below, optimal primer annealing temperature and PCR cycle number need to be standardized for each primer pair per target region. Please refer to **steps 5 – 7** of this protocol.

50. Amplify the area surrounding the specific SGE target region from the collected gDNA pool.
  a. Prepare a PCR reaction master mix as indicated below.
    ***Note:*** Set up three 50 μL PCR reactions for each time point-replicate**.** Each 50 μL reaction should contain 1 μg of gDNA, resulting in 3 μg of gDNA per time point-replicate (which equates to roughly 1 million haploid genomes, with a mass of ∼3 pg).
    **Table:** PCR reaction master mix to amplify edited gDNA enrichment—Using Primer Set 6

Reagent	Amount
Time point-replicate gDNA	X μL (1 μg)
gdamp_F (10 μM)	1.5 μL
gdamp_R (10 μM)	1.5 μL
KAPA HiFi Hotstart ReadyMix (2X)	25 μL
ddH_2_O	Complete to 50 μL
  b. Run a PCR following the conditions below.
    **Table:** PCR cycling conditions for amplifying edited gDNA enrichment—Using Primer Set 6

Steps	Temperature	Time	Number of cycles
Initial Denaturation	95°C	3 min	1
Denaturation	98°C	20 s	20 - 25 cycles
Annealing	58°C–65°C	15 s	
Extension	72°C	1 min 30 s	
Final extension	72°C	1 min 30 s	1
Hold	4°C	forever
51. Pool the 3 x 50 μL reactions per time point-replicate into a 1.5 mL Eppendorf tube.
52. Purify each sample using the QIAquick PCR purification Kit (QIAGEN), following the manufacturer’s instructions.
  ***Note:*** Total volume per sample: 150 μL.
  a. Elute the DNA in 40 μL EB Buffer.
53. Perform an enzymatic PCR cleanup with ExoI (to remove ssDNA & primers).
  a. Prepare a master mix, as described in the materials and equipment section under “Reaction setup for ExoI enzymatic cleanup of the PCR product of Primer Set 6”.
  b. Vortex and mix the reaction.
  c. In a thermocycler, incubate the reaction at 37°C for 20 min followed by 80°C for 20 min.
54. Purify the ExoI-digested PCR product(s) of Primer Set 6 via MinElute columns (QIAGEN), following the manufacturer’s instructions.
  a. Elute the DNA in 15 μL of EB Buffer.
  b. Measure the DNA concentration of the purified PCR product(s) of Primer Set 6.
  c. Dilute samples to 10 ng/μL in EB Buffer.

**Pause point:** The PCR product can be stored at −20°C for long-term storage.

### Primer design for PCR for sequence adapter addition/amplification of edited gDNA regions—Primer Set 7

**Timing: 20 min, depending on number of samples**

The following steps outline the primer design for Primer Set 7, as shown in the key resources table of this protocol. This primer set further amplifies the specific target region from the Primer Set 6 PCR product and incorporates Illumina adapters (for sequencing), allowing for the addition of individual sequencing barcodes in subsequent steps.

55. Design Primer Set 7 using any preferred primer design software.
  ***Note:*** In this protocol, we used the ‘Primer Design’ function within Geneious Prime, a modified version of Primer 3.
  a. Product size should between ∼245-300 bp, the same as for the individual SGE target regions.
  b. The primers flank the target region, both facing inwards, orientated 5'>3'.
  c. The sequencing forward primer should be designed as: Indexing barcode dependent adaptors + ‘Primer Set 2_F’, 5' and 3', respectively.
  d. The sequencing reverse primer should be designed as: Indexing barcode dependent adaptors + ‘Primer Set 2_R’, 5' and 3', respectively.

### PCR for sequence adapter addition/amplification of edited gDNA regions using Primer Set 7

**Timing: 2 days, depending on number of target regions**

In this step a PCR is performed that uses Primer Set 7, as shown in the key resources table of this protocol, to amplify the edited target-specific gDNA regions. Please refer to Figure 7B for an illustration of this process.

***Note:*** Before performing the PCR below, optimal primer annealing temperature and PCR cycle number need to be standardized for each primer pair per target region. Please refer to **steps 5 – 7** of this protocol.

56. Amplify the target-specific gDNA region from ‘Primers Set 7’ PCR product.
  a. Prepare a PCR reaction master mix as indicated below.
    ***Note:*** Set up three 50 μL PCR reactions for each time point-replicate**.** Each 50 μL reaction should contain 25 ng of DNA, resulting in 75 ng of DNA per time point-replicate.
    **Table:** PCR reaction master mix for edited target-specific region amplification and Illumina adapter addition—Using Primer Set 7

Reagent	Amount
PCR product of Primer Set 6 (10 ng/μL stock)	2.5 μL (25 ng)
sequencing_F (10 μM)	1.5 μL
sequencing_R (10 μM)	1.5 μL
KAPA HiFi HotStart ReadyMix (2X)	25 μL
ddH_2_O	Complete to 50 μL
    **Table:** PCR cycling conditions for edited target-specific region amplification and Illumina adapter addition—Using Primer Set 7

Steps	Temperature	Time	Number of cycles
Initial Denaturation	95°C	3 min	1
Denaturation	98°C	20 s	6-15 cycles
Annealing	58°C–65°C	15 s	
Extension	72°C	30 s	
Final extension	72°C	30 s	1
Hold	4°C	forever
  b. Run a PCR following the conditions below.
57. Pool the 3 x 50 μL reactions per time point-replicate into a 1.5 mL Eppendorf tube.
58. Purify each sample using the QIAquick PCR purification Kit (QIAGEN), following the manufacturer’s instructions.
  ***Note:*** Total volume per sample: 150 μL.
  a. Elute the DNA in 30 μL EB Buffer.
59. Perform an enzymatic PCR cleanup with ExoI (to remove ssDNA & primers).
  a. Prepare a master mix, as described in the materials and equipment section under “Reaction setup for ExoI enzymatic cleanup of the PCR product of Primer Set 7”.
  b. Vortex and mix the reaction.
  c. In a thermocycler, incubate the reaction at 37°C for 20 min followed by 80°C for 20 min.
60. Purify the ExoI-digested PCR product(s) of Primer Set 7 via MinElute columns (QIAGEN), following the manufacturer’s instructions.
  ***Note:*** After adding PB Buffer, add 3 μL of 3M NaOAC pH 5.2 (to adjust the pH) to each sample.
  a. Elute the DNA in 12 μL of EB Buffer.
  b. Measure the DNA concentration of the purified PCR product(s) of Primer Set 7.
  c. Dilute samples to 5 ng/μL in EB Buffer.
    **Pause point:** The PCR product can be stored at −20°C for long-term storage.

### Indexing PCR to add individual barcodes to samples for NGS using Primer Set 8

**Timing: 1 day, depending on number of samples**

In this step a PCR is performed that uses Primer Set 8, as shown in the key resources table of this protocol, which allow for the addition of individual sequencing barcodes to each time point-replicate. Please refer to Figure 7C for an illustration of this process.

61. Add indexing barcode to each time point-replicate sample.
  a. Prepare a PCR reaction master mix as indicated below.
    **CRITICAL:** Use individual indexing primers for each sample. No design or optimizations are needed these primers. The primers are ordered either combined pre-mixed or can be added to reactions individually.
    **Table:** Indexing PCR reaction master mix—Using Primer Set 8

Reagent	Amount
PCR product of Primer Set 7 (5 ng/μL stock)	5 μL (25 ng)
IDT_indexing_F (10 μM)	1.5 μL
IDT_indexing_R (10 μM)	1.5 μL
KAPA HiFi HotStart ReadyMix (2X)	25 μL
ddH_2_O	Complete to 50 μL
  b. Run PCR following the below conditions
    **Table:** Indexing PCR cycling conditions—Using Primer Set 8

Steps	Temperature	Time	Number of cycles
Initial Denaturation	95°C	3 min	1
Denaturation	98°C	20 s	7 cycles
Annealing	59°C	15 s	
Extension	72°C	30 s	
Final extension	72°C	30 s	1
Hold	4°C	forever
62. Purify the PCR products of Primer Set 8 using Ampure XP beads.
  ***Note:*** This process can be done either in a Cornig 96-well plate (round bottom) or in individual 1.5 mL Eppendorf tubes.
  **CRITICAL:** Leave Ampure XP beads at 20°C–25°C for ∼30 min prior to start.
  a. Vortex Ampure XP beads to resuspend.
  b. Add 45 μL (0.9X) of resuspended beads to 50 μL indexed PCR reaction. Mix well by pipetting up and down at least 10 times.
    ***Note:*** Be careful to expel all the liquid out of the tip during the last mix.
  c. Incubate samples at 20°C–25°C for 15 min.
  d. During the incubation, prepare fresh 80% ethanol, ensuring enough volume to wash each sample with 400 μL.
  e. After the incubation, place the plate on a magnetic stand to separate the beads from the supernatant. After 5 min, carefully remove 30 μL of the supernatant from the top.
    ***Note:*** The removed supernatant should look clear.
  f. Seal and spin the plate at 1000 RPM for 20 s.
    ***Note:*** Most of the beads should be at the bottom and the solution should look clear.
  g. Remove the seal and place on the magnetic rack. After 5 min, remove all the solution.
    ***Note:*** Repeat **step 62f**, if necessary.
  h. Add 200 μL of the 80% freshly prepared ethanol to the plate while in the magnetic stand.
    **CRITICAL:** Add to the side opposite the pellet so as not to disturb the pellet.
  i. Incubate at 20°C–25°C for 30 s, and then carefully remove and discard the supernatant.
    **CRITICAL:** Be careful not to disturb the beads that contain the DNA.
  j. Repeat **Step 62h** and **i** for a total of two washes.
  k. Seal and spin the plate at 1000 RPM for 20 s.
  l. Remove the seal and place it on the magnetic rack. Remove traces of ethanol with a long reach p10 pipette tip.
    ***Note:*** Repeat **step 62k**, if necessary.
  m. Air dry the beads for up to 5 min while the plate is on the magnetic stand.
    **CRITICAL:** Do not over-dry the beads. This may result in lower recovery of DNA. Elute the samples when the beads are still dark brown and glossy looking, but when all visible liquid has evaporated. When the beads turn lighter brown and start to crack, they are too dry.
  n. Remove the plate from the magnetic stand. Elute the DNA by adding 33 μL of EB Buffer.
  o. Mix well by pipetting up and down 10 times. Incubate at 20°C–25°C for 10 min.
  p. Place the plate on the magnetic stand. After 5 min (or when the solution is clear) transfer 30 μL to a new plate or a new 1.5 mL Eppendorf tube per time point-replicate.
    ***Note:*** Ensure that you did not take out any beads, if possible.
  q. Measure the DNA concentration of the bead-purified DNA.
    **Pause point:** The PCR product can be stored at −20°C for long-term storage.
63. Control gel of bead-purified DNA samples.
  a. For each SGE edited gDNA library, prepare 15 μL of 10 ng/μL SGE edited gDNA library with EB Buffer.
  b. Run 8 μL of the 10 ng/μL SGE edited gDNA library on a 2% agarose-TAE gel at 120 V for 30 min.
    i. As a control, run 80 ng of the purified PCR product of Primer Set 7 on the gel (∼245-300 bp) to confirm the expected band size increase and assess relative concentration.
  c. Confirm the fragment size, clarity and intensity on the gel.
    ***Note:*** You should see a single band at ∼350-400 bp (Figure 8). Each SGE edited gDNA library should exhibit similar band intensity if DNA quantification was performed correctly.
    ***Note:*** If some bands on the gel exhibit significant variation in intensity compared to other bands, the DNA concentration of these samples should be re-evaluated to ensure that uniform concentrations (10 ng/μL) across all samples are used for subsequent steps, this helps to normalize seqeuncing depth in the pooled NGS library.

**Figure 8:**
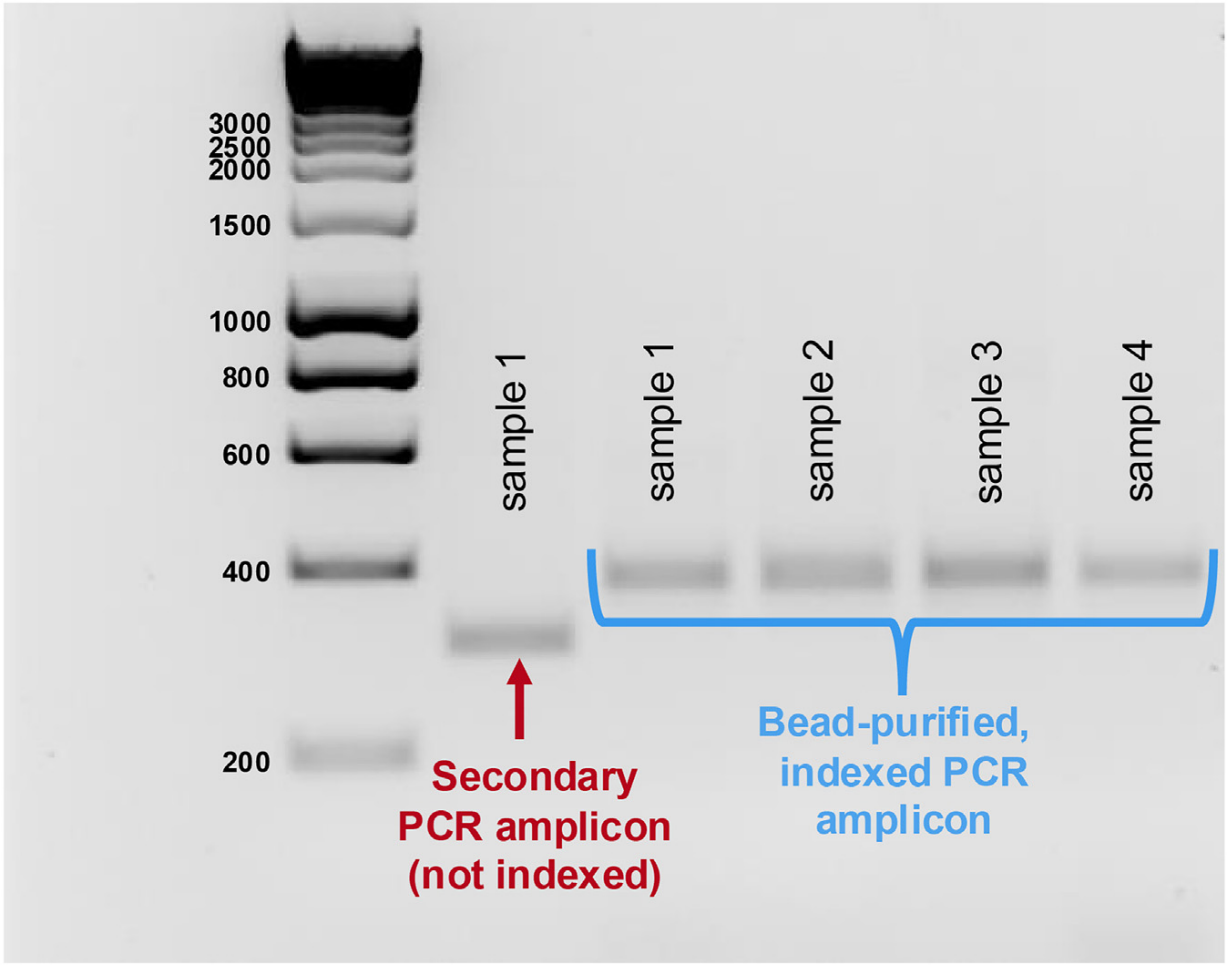
Control gel electrophoresis of bead-purified samples prior to sequencing submission 80 ng of DNA sample and an appropriate volume of loading dye were used for gel electrophoresis. Lane 1: 1 kb HyperLadder. Lane 2: Sample 1 following secondary PCR (non-indexed), with an expected fragment size between ∼245-300 base pair (bp). Lane 3: Sample 1 after indexing in the PCR round, showing an expected larger fragment size due to the addition of a unique barcode. Lanes 4–6: Three additional indexed sample examples, which show larger fragment sizes compared to the secondary PCR product. All band intensities are within an acceptable range, and no non-specific bands are observed.
  d. **Optional step:** Samples can also be analyzed using the Agilent TapeStation system to assess the integrity, size, and concentration of samples.
  e. Pool 5 μL of each 10 ng/μL SGE edited gDNA library into one 1.5 mL Eppendorf tube to created a pooled NGS library.
  f. Sequence the samples using NGS at sufficient depth to achieve roughly 500x variant coverage. Read allocation will depend on specific platform and number of NGS libraries in the pool, please refer to Waters et al^2^ for an example.
64. Analyze data to calculate the depletion kinetics of the variants within target regions.

***Note:*** SGE variant quantification, functional scoring, classifications, and data analysis have been previously conducted for the *DDX3X*,^1^*BAP1*,^2^ and *RAD51C*^3^ genes, providing established examples for the analysis of SGE datasets.

## Expected outcomes

This protocol facilitates the generation of high-throughput mutational data, which can be analyzed using various approaches. Comprehensive methodologies for SGE data analysis, including functional score derivation, for the *DDX3X*,^1^ *BAP1*,^2^ and *RAD51C*^3^ genes are available for future reference. Briefly, SGE high-throughput sequencing data is processed into read counts, with SGE edited gDNA library-specific counts generated for each indexed time point and replicate. These counts are subsequently aggregated, quality-checked, and integrated with SGE HDR template library counts into a data frame. The resulting counts are utilized as input for DESeq2^18^ to compute log_2_-fold changes between time points, enabling the assessment of variant dynamics over time. Variants are then correlated with the mutational consequences incorporated into the SGE oligo library design, followed by additional quality control steps. Functional scores for variants can be derived using analytical frameworks described in prior studies.^1^^,^^2^^,^^3^

The overarching goal of SGE data is to produce clinically relevant variant functional scores, thereby advancing efforts to resolve variants of uncertain significance (VUS). This contributes to improved clinical management, personalized medicine, and genetics research.

## Limitations

### Cell essentiality in HAP1-A5 cells

An analysis of the Cancer Gene Census identified that 59% of genes are predicted to exhibit a strong growth phenotype in HAP1 cells.^17^ However, not all genes are essential. Notably, 46% of non-essential genes in HAP1 cells, including clinically relevant oncogenes, require alternative phenotyping approaches to the here presented protocol, for accurate functional characterization. Although we have no direct evidence that cell context influences editing readout at the gene level, factors such as cell type, the patient background from which HAP1 cells were derived, and the conditions in which the cells are grown could all impact the functional readout.

### Potential PCR optimizations per target region

Despite the troubleshooting strategies and optimization suggestions provided in this protocol, researchers may still encounter challenges with specific primer sets. These difficulties may arise due to issues such as primer design, template quality or quantity, reaction conditions, non-specific binding, or polymerase selection. If primer-related issues persist, it is recommended to redesign the primers by selecting a new primer pair with a more suitable annealing temperature, balanced GC content, and reduced likelihood of template mismatches.

## Troubleshooting

### Problem 1

SGE primers (Primer Set 2, Primer Set 4, or Primer Set 7) produce non-specific results, such as multiple bands or smeared gel patterns.

### Potential solution

First, experiment with different annealing temperatures and consider using alternative polymerases, such as the Q5 High-Fidelity DNA Polymerase, with and without DMSO. If these adjustments do not improve results, redesign the primers for the target exon, shifting the binding sites while maintaining the same insert size. Additionally, ensure the target region remains relatively centralized to optimize amplification efficiency.

### Problem 2

KAPA Hifi HotStart ReadyMix 2X is not performing well during SGE post-screen sample preparation for NGS.

### Potential solution

Consider using an alternative polymerase, such as repliQa HiFi ToughMix, which may offer better performance under the given conditions. When switching polymerases, it is essential to optimize the reaction conditions and conduct comparative testing to select the polymerase that provides the best results.

### Problem 3

Target regions are difficult to amplify during SGE post-screen sample preparation for NGS.

### Potential solution

A first step might be to redesign Primer Set 6 as there is flexibility with these primers as they do not inform the experimental design beyond the need for one of the primers to be outside of cloned homology arm regions. An alternative might be to implement a PCR touchdown protocol, as shown below, where the initial annealing temperature is set high and gradually reduced over successive cycles. This approach improves primer specificity and enhances DNA amplification efficiency, as supported by previous studies. However, this solution may lead to less representation of editing as amplification potentially proceeds more from amplicons than from gDNA. This should only be used as a last resort and carefully checked for quality.^19^^,^^20^

**Table:** PCR cycling conditions for Touchdown PCR

Step	Temp	Time	
1	95°C	3 min	
2	98°C	20 s	
3	67 (−1/cycle) °C	15 s	
4	72°C	1 min 30 s	

**Back to step 2 for 11 total cycles**			

5	98°C	20 s	
6	57°C	15 s	
7	72°C	1 min 30 s	

**Back to step 5 for 13 total cycles**			

5	72°C	1 min 30 s	

### Problem 4

Endura cell transformation for cloning SGE HDR template libraries does not produce sufficient colony growth/coverage (i.e. 1% of transformation contains <500 colonies = <50x cloning coverage for SGE variant libraries containg 1000 variants).

### Potential solution

First, repeat the transformation, performing 2–3 reactions per sample and pooling them. If unsuccessful, run a gel for the NEBuilder ligation reaction from **step 24** to verify band size. If the size is incorrect, repeat the ligation. If the problem persists after confirming a successful ligation, try one or more of the following: use a higher concentration of the amplified 'target specific SGE variant oligonucleotide library' and 'linearized wild-type homology region' (e.g. 75ng and 150ng, respectively) for the ligation or use a different strain of competent cells (i.e., Stellar chemically competent cells). If necessary, re-prepare individual fragments and follow the protocol closely.

## Resource availability

### Lead contact

Further information and requests for resources and reagents should be directed to and will be fulfilled by the lead contact, Dr. Andrew J. Waters (aw28@sanger.ac.uk).

### Technical contact

Questions about the technical specifics of performing the protocol should be directed to the technical contacts, Dr. Andrew J. Waters (aw28@sanger.ac.uk) and Sofia Obolenski (so16@sanger.ac.uk).

### Materials availability

This study did not generate new unique reagents.

### Data and code availability

This study did not generate/analyze datasets/code.

## Acknowledgments

This research was funded by the Cancer Research UK CG-MAVE programme and the Wellcome Trust (grant no. 220540/Z/20/A, “Wellcome Sanger Institute Quinquennial Review 2021–2026”).

## Author contributions

S.O. and R.O.-L. optimized the protocol, performed the experiments, and prepared the manuscript. D.S. performed the experiments and optimized the Endura cell transformations. D.J.A. designed the experiments and prepared the manuscript. A.J.W. designed the experiments, optimized the protocol, performed the experiments, and prepared the manuscript. All authors reviewed, approved, and commented on the manuscript.

## Declaration of interests

The authors declare no competing interests.
